# Systematic Review with Meta-Analysis: The Effects of Probiotics in Nonalcoholic Fatty Liver Disease

**DOI:** 10.1155/2019/1484598

**Published:** 2019-12-11

**Authors:** Meng-Wei Xiao, Shi-Xin Lin, Zhao-Hua Shen, Wei-Wei Luo, Xiao-Yan Wang

**Affiliations:** ^1^Department of Gastroenterology, Third Xiangya Hospital, Central South University, Changsha 410013, China; ^2^Hunan Key Laboratory of Nonresolving Inflammation and Cancer, Changsha 410013, China; ^3^Department of Gynecology and Obstetrics, Third Affiliated Hospital of Nanchang University, Nanchang 330000, China

## Abstract

**Background and Aims:**

Probiotics was considered as a potential therapy for nonalcoholic fatty liver disease (NAFLD) without approval and comprehensive assessment in recent years, which call for a meta-analysis.

**Methods:**

We performed electronic and manual searches including English and Chinese databases published before April 2019, with the use of mesh term and free text of “nonalcoholic fatty liver disease” and “probiotics.” Clinical trials evaluating the efficacy of probiotic therapy in NAFLD patients were included according to the eligibility criteria. With the use of random effects models, clinical outcomes were presented as weighted mean difference (WMD) with 95% confidence interval (CI), while heterogeneity and meta-regression were also assessed.

**Results:**

28 clinical trials enrolling 1555 criterion proven NAFLD patients with the use of probiotics from 4 to 28 weeks were included. Overall, probiotic therapy had beneficial effects on body mass index (WMD: -1.46, 95% CI: [-2.44, -0.48]), alanine aminotransferase (WMD: -13.40, 95% CI: [-17.03, -9.77]), aspartate transaminase (WMD: -13.54, 95% CI: [-17.86, -9.22]), gamma-glutamyl transpeptidase (WMD: -9.88, 95% CI: [-17.77, -1.99]), insulin (WMD: -1.32, 95% CI: [-2.43, -0.21]), homeostasis model assessment-insulin resistance (WMD: -0.42, 95% CI: [-0.73, -0.12]), and total cholesterol (WMD: -15.38, 95% CI: [-26.50, -4.25]), but not in fasting blood sugar, lipid profiles, or tumor necrosis factor-alpha.

**Conclusion:**

The systematic review and meta-analysis support that probiotics are superior to placebo in NAFLD patients and could be utilized as a common complementary therapeutic approach.

## 1. Introduction

Nonalcoholic fatty liver disease (NAFLD), which characterized by the accumulation of lipid in liver parenchyma without obvious alcohol consumption, is a clinical syndrome of chronic liver disease scoping from simple steatosis, nonalcoholic steatohepatitis (NASH) to cirrhosis [1]. Nowadays, NAFLD has become the most common liver disease affecting adults and children in the world, with 52.34 per 1000 person-years overall global prevalence rate [2, 3]. It is reported that hepatocellular carcinoma is closely associated with NAFLD, leading to a higher mortality rate of NAFLD patients than the general population [4]. With the rapidly rising of morbidity, NAFLD imposes a major threat to the health of human and has become a worldwide public health problem [5].

However, no standard pharmacologic therapy is available for NAFLD currently. In view of the burden to NAFLD, a pressing need in pharmacologic treatment options is to be solved for this patient population [6]. Some evidences suggested that gut-liver axis is closely associated with NAFLD. There are over 10000 microbes that live in a symbiotic relationship with human body in intestinal tract and could influence the host in a variety of ways. For example, endotoxin produced by intestinal bacteria could be phagocyted by the Kupffer cells in the liver via blood circulation and therefore lead to a constantly expose, which conduces to the progression of liver inflammation [7]. Consequently, a supplement of probiotics to regulate the imbalanced intestinal flora and reduce the production of detrimental metabolite has a potential value in the treatment of NAFLD.

Lots of RCTs surrounding probiotics and NAFLD have been published in recent year, but efficacies of probiotics remain controversial. We therefore performed a systematic literature search and meta-analysis to provide an overview of the currently available evidence for the efficacy of probiotics in the treatment of NAFLD patients.

## 2. Materials and Methods

This systematic review was conducted according to the Preferred Reporting Items for Systematic Reviews and Meta-Analyses (PRISMA) guidelines [8]. Briefly, clinical trials assessing the effects of probiotics versus a control group were included. Two investigators independently performed following data extraction, risk of bias, meta-analyses, and Grading of Recommendations Assessment, Development and Evaluation (GRADE) scoring, with divergences resolved by a third investigator.

### 2.1. Date Sources and Literature Search

Reports in English and Chinese languages published from the establishment of each database to April 2019 were reviewed. The English databases included PubMed, Embase, Cochrane Library, Web of Science, and OVID. The Chinese databases included China National Knowledge Infrastructure, VIP Database for Chinese Technical Periodicals, China Biology Medicine disc, and Wanfang Database. The literature searches were performed by two reviewers independently. Mesh term and free text including “nonalcoholic fatty liver disease” and “probiotics” were used. The full search strategy is available in supplementary data. The searches were performed without limiting the types of studies to maximize scope. We also searched abstracts and references from bibliographies of relevant studies, review articles, and meta-analyses for additional items manually.

### 2.2. Study Selection

Two reviewers screened the titles and abstracts of the identified papers to further check the eligibility criteria independently. The full texts of the studies were assessed when abstracts could not provide clear information. To be eligible for inclusion, studies testing the use of probiotics in the treatment of NAFLD were included. There was no specific restriction (for example, age or sex). The PICOS criteria for inclusion and exclusion of studies is shown in Table 1.

Studies were considered eligible if they met the following criteria: (a) studies testing the effects of probiotics in the treatment of NAFLD patients; (b) patients with NAFLD should diagnosed on the basis of radiological/histological evidence of fatty liver, with daily alcohol intake restriction (less than 14 standard drinks in women and 21 standard drinks in men per week) [2]; (c) randomized and controlled design with the probiotic group and control group; (d) cointerventions were also considered eligible when they used both intervention arms equally; (e) studies which directly evaluated the effect of probiotics based on any method of outcome measures; (f) studies were written in English or Chinese; and (g) all data needed are available.

Studies were excluded if they (a) were case reports, reviews, or letters; (b) were only published as conference abstracts or contained no original data; (c) contained duplicate data already published; (d) performed no control group; and (e) contained patients with other causes of hepatic steatosis.

### 2.3. Data Extraction

All data were independently abstracted in duplicate by two reviewers using a predefined data form, with disagreements resolved with a third reviewer. Information including study design, characteristics, population, details of intervention, and control were extracted. Mean and standard deviation (SD) of each endpoint were either extracted or calculated from data in each study.

### 2.4. Risk of Bias

We used funnel plots to provide a visual assessment of the association between treatment estimate and study size. Egger's tests were performed to assess the asymmetry of funnel plots, with significant publication bias defined as *P* value < 0.05 [9]. The trim and filling computation was conducted to estimate the robustness of results [10]. Jadad scale was applied to assess quality of randomized controlled trials, while RCT scoring ≥3 was considered acceptable [11]. The risk of bias associated with the RCTs literature risk was assessed by the Cochrane Risk of Bias Tool [12] in the Cochrane Handbook for Systematic Reviews of Interventions in RevMan software (RevMan version 5.3).

### 2.5. Statistical Analysis

Primary outcomes were liver-related outcomes, for example, serum level of alanine aminotransferase (ALT), aspartate transaminase (AST), and gamma-glutamyl transpeptidase (GGT). Secondary outcomes included metabolic outcomes, for example, change in body mass index (BMI), fasting blood sugar (FBS), insulin, homeostasis model assessment-insulin resistance (HOMA-IR), high-density lipoprotein cholesterol (HDL-C), low-density lipoprotein cholesterol (LDL-C), triglycerides (TG), total cholesterol (TC), and tumor necrosis factor-alpha (*Tnf-α*).

For the meta-analysis, we performed comparisons with a random effects model because they are more conservative and have better properties in the presence of heterogeneity [13]. The differences of measured continuous parameters were calculated and analyzed using weighted mean difference (WMD/MD) changes from baseline along with the 95% confidence intervals (CIs). A statistically significant *P* value was based on <0.05. We assessed heterogeneity between the studies using the *I*^2^ statistic, while low, moderate, and high levels of heterogeneity approximately correspond to *I*^2^ values of 25%, 50%, and 75%, respectively, and *I*^2^ < 50% was considered as acceptable heterogeneity [14]. Data of each indicator was pooled and shown as forest plot. Subgroup analyses were performed mainly according to the probiotic strains taken by patients, including *Lactobacillus* spp. subgroup, *Bifidobacterium* spp. subgroup, *Lactobacillus* spp.+*Bifidobacterium* spp. subgroup, *Lactobacillus* spp.+*Bifidobacterium* spp.+others subgroup, and others subgroup. Meta-regression was performed to explore possible sources of heterogeneity (i.e., the age and regions of patients, the doses and durations of interventions, and the details of additional treatment) which could lead to confounding in our analysis. To test robustness of the association, sensitivity analysis was also employed. We examined the influence of a single study on the combined risk estimates by omitting one study and analyzing the remainders in each turn [15]. We also conducted separate meta-analyses and subgroup analysis based on studies that used different probiotic strains.

Review Manager version 5.3 was selected to analyze the tests for the forest plots, subgroup analysis, and quality assessment, while the risk of publication bias and meta-regression analysis were performed by Stata version 12.0. Egger's tests; sensitivity analysis and meta-regression were only performed for items which include over ten studies.

### 2.6. Quality of Evidence

Additionally, we assessed the strength of evidence using the GRADE framework with GRADEprofiler version 3.6 [16]. More concretely, outcomes were graded according to risk of bias, consistency and directness of results, precision, publication bias, and magnitude. Finally, evidences were defined as high, moderate, low, and very low quality.

## 3. Results

### 3.1. Characteristics of the Retrieved Studies and Patients

**Figure 1:**
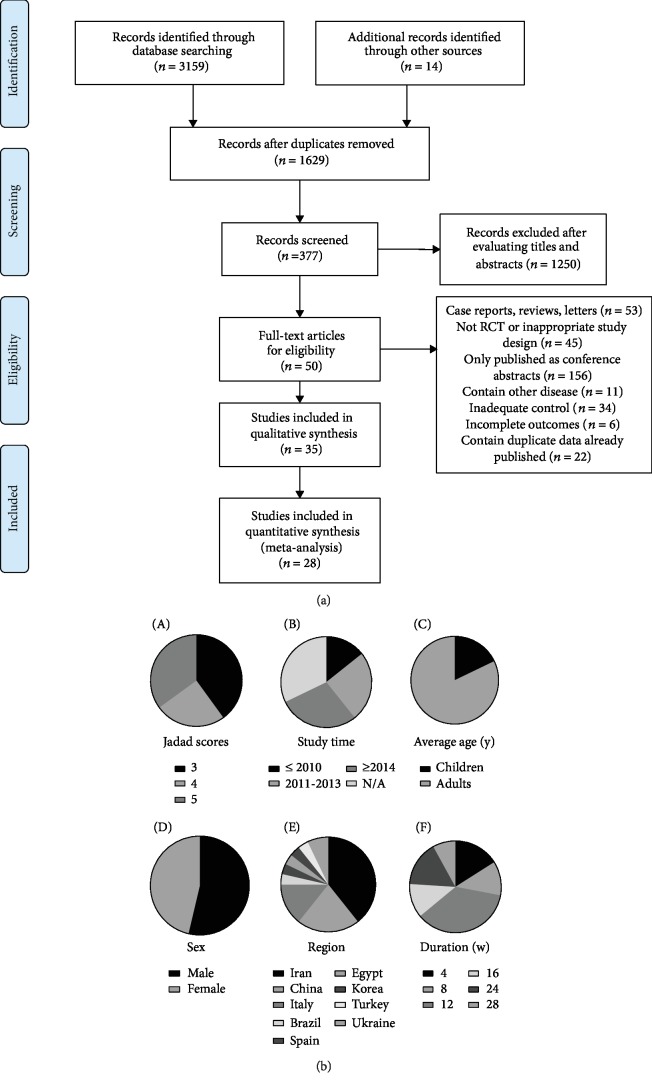
(a) Flow diagram of study selection. (b) Analysis of the general information in included studies.

**Figure 2:**
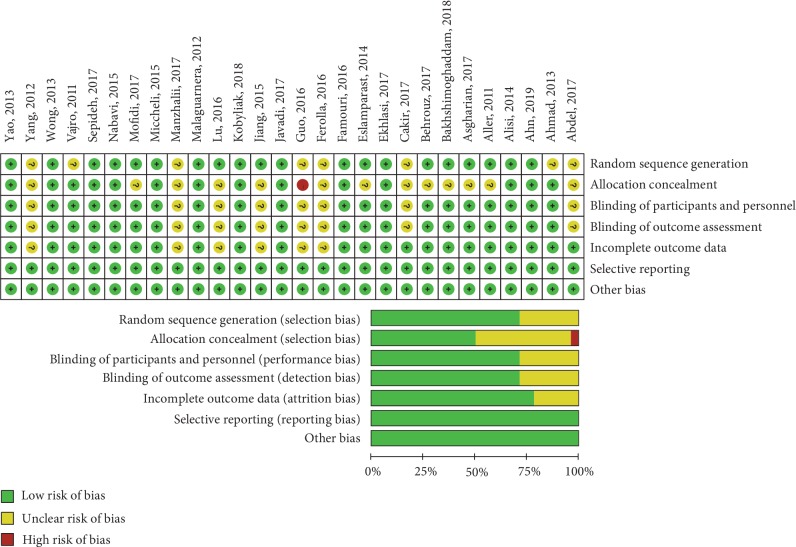
Analysis for risk of bias. 28 studies were analyzed for a variety of bias using the tools in RevMan software.

The electronic searches yielded 3159 items from databases mentioned above, and 14 additional records were identified through other sources. After reviewing each publication, we selected 28 studies according to inclusion and exclusion criteria (the included studies are shown as references [17–44]). A flow chart for the literature retrieval and screening is presented in Figure 1(a). The studies included predominantly RCTs.  Table 2 demonstrates the available detailed information of trials. They scored well in terms of adequate descriptions of selection criteria and the availability of clinical data (Figure 1(b) A). Eight (29%) studies were published after 2014 (Figure 1(b) B). These twenty-eight studies included a total of 1555 NAFLD patients, within 824 (111 children) in the probiotic group and 731 (112 children) in the control group (Figure 1(b) C). The distribution of sex is shown in Figure 1(b) D. Most of the included patients were from Iran (11, 39%), China (6, 21%), and Italy (4, 14%) (Figure 1(b) E). And the durations of probiotics taken range from 4 to 28 weeks (Figure 1(b) F). The recruited studies were further subjected to risk analyses for bias, while Figure 2 is a summary of the assessment results. Most of the studies were in low categories for risk of bias, random sequence generation (20/28, 71%), incomplete outcome data (22/28, 79%), and allocation concealment (13/28, 76%), blinding of outcome assessment (20/28, 71%).

### 3.2. Impact of Probiotics on BMI in NAFLD Patients

**Figure 3:**
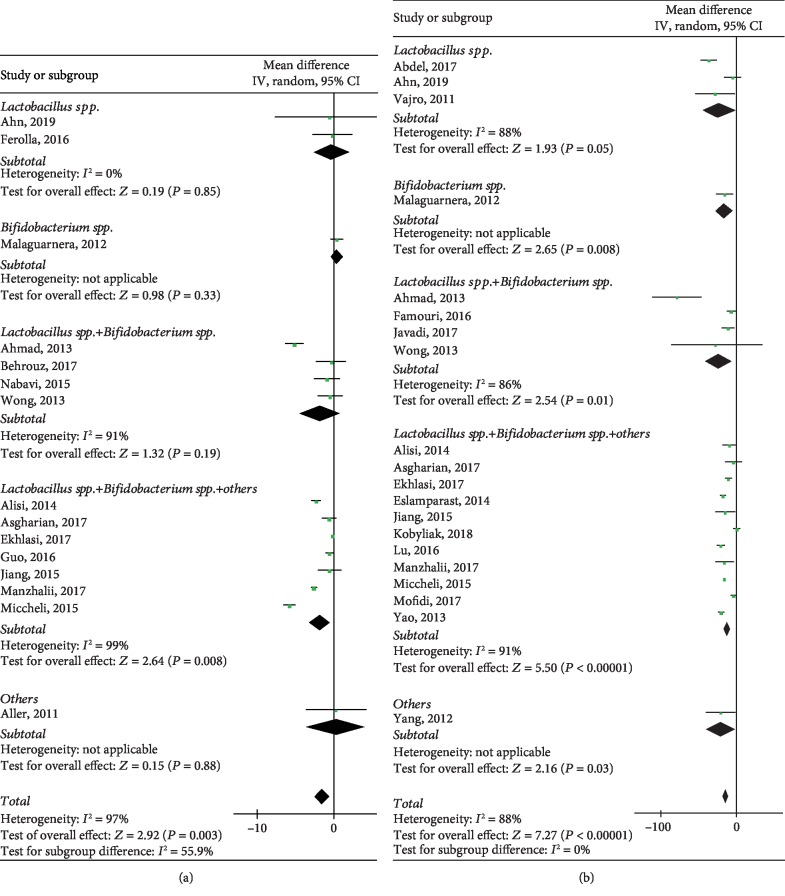
Forest plots of comparison for the effects of probiotics in NAFLD patients, showing (a) body mass index (BMI) and (b) alanine aminotransferase (ALT).

The effect of probiotics on BMI was studied in 15 of the identified studies. Results showed that the trend was significantly associated with probiotics (WMD: -1.46, 95% CI: [-2.44, -0.48], *P* = 0.003) with obvious heterogeneity (*P* < 0.00001, *I*^2^ = 97%), including 818 individuals. Sensitivity analyses corroborated a good robustness of the association, without evidence of publication bias (*P* = 0.129) ([Supplementary-material supplementary-material-1]). However, our subgroup analysis which according to the probiotic strains taken by patients indicated negative results except *Lactobacillus* spp.+*Bifidobacterium* spp.+others subgroup (*P* = 0.008). Besides, heterogeneity remained significant in *Lactobacillus* spp.+*Bifidobacterium* spp. subgroup (*I*^2^ = 91%) and *Lactobacillus* spp.+*Bifidobacterium* spp.+others subgroup (*I*^2^ = 99%), which means probiotic strains could not explain the source of heterogeneity, as shown in Figure 3(a).

### 3.3. Impact of Probiotics on Liver Functions in NAFLD Patients

#### 3.3.1. ALT

Data regarding ALT extracted from 20 studies included 1116 individuals, and the analyses showed a significant association between the probiotic group and placebo group (WMD: -13.40, 95% CI: [-17.03, -9.77], *P* < 0.00001) in 20 heterogeneous studies (*P* < 0.00001, *I*^2^ = 94%). There was no publication bias (*P* = 0.135) and the trim and filling computation justified good robustness ([Supplementary-material supplementary-material-1]).  Figure 3(b) shows a significant result in each subgroup (*P* = 0.05, *P* = 0.008, *P* = 0.001, *P* < 0.00001, *P* = 0.03), but they are heterogeneous (*I*^2^ = 88%, *I*^2^ = 86%, *I*^2^ = 91%).

#### 3.3.2. AST

**Figure 4:**
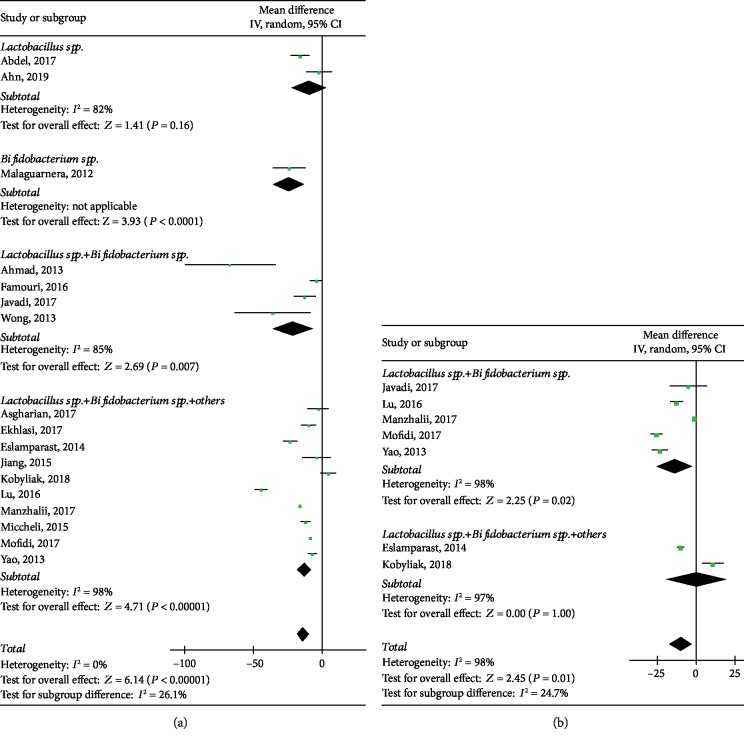
Forest plots of comparison for the effects of probiotics in NAFLD patients, showing (a) aspartate transaminase (AST) and (b) gamma-glutamyl transpeptidase (GGT).

The analyses of AST, which were performed based on pooled data extracted from 17 heterogeneous studies (*P* < 0.00001, *I*^2^ = 96%), demonstrated that the use of probiotics could reduce AST level significantly among 992 patients (WMD: -13.54, 95% CI: [-17.86, -9.22], *P* < 0.00001). There was no obvious publication bias (*P* = 0.639) by Egger's tests ([Supplementary-material supplementary-material-1]). Specifically, heterogeneity was restricted to *Lactobacillus* spp. (*P* = 0.16, *I*^2^ = 82%), *Lactobacillus*spp.+*Bifidobacterium* spp. subgroup (*P* = 0.007, *I*^2^ = 85%), and *Lactobacillus* spp.+*Bifidobacterium* spp.+others subgroup (*P* < 0.00001, *I*^2^ = 98%), as shown in Figure 4(a).

#### 3.3.3. GGT

Seven studies investigated GGT between the interventional group and the control group. Results showed that GGT was significantly associated with probiotic (WMD: -9.88, 95% CI: [-17.77, -1.99], *P* = 0.01) with obvious heterogeneity (*P* < 0.00001, *I*^2^ = 98%), including 488 individuals. The heterogeneity remained significant in the *Lactobacillus* spp.+*Bifidobacterium* spp. subgroup (*P* = 0.02, *I*^2^ = 98%), but in *Lactobacillus* spp.+*Bifidobacterium* spp.+others subgroup, there is no significant difference with high heterogeneity (*P* = 1.00, *I*^2^ = 97%).

### 3.4. Impact of Probiotics on Glycemic Indices in NAFLD Patients

#### 3.4.1. FBS

**Figure 5:**
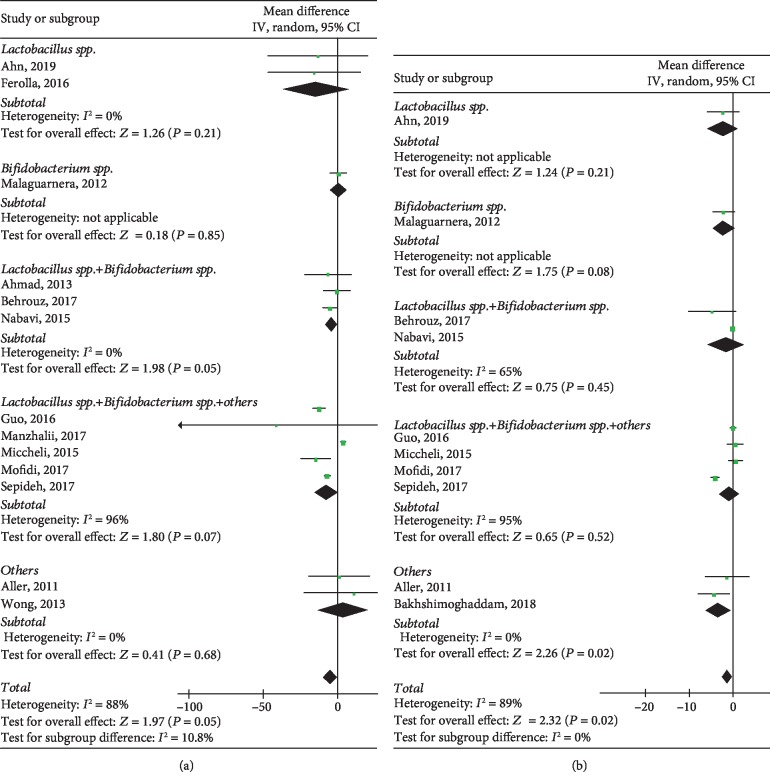
Forest plots of comparison for the effects of probiotics in NAFLD patients, showing (a) fasting blood sugar (FBS) and (b) insulin.

In the case of FBS, there was no statistical difference between the probiotic and control groups (WMD: -4.98, 95% CI: [-9.95, -0.02], *P* = 0.05) in 13 heterogeneous studies (*P* < 0.00001, *I*^2^ = 88%), including 711 individuals. The trim and filling computation showed a robust result with the absence of publication bias (*P* = 0.413) ([Supplementary-material supplementary-material-1]). The subgroup analysis was able to partly explain the heterogeneity. However, there was no difference in statistical significance, although we conducted the subgroup analysis (*P* = 0.21, *P* = 0.85, *P* = 0.05, *P* = 0.07, *P* = 0.68), as shown in Figure 5(a).

#### 3.4.2. Insulin

Ten studies reported on insulin between the interventional group and the control group.  Figure 4(b) shows that insulin was significantly associated with probiotics (WMD: -1.32, 95% CI: [-2.43, -0.21], *P* = 0.02) with obvious heterogeneity (*P* < 0.00001, *I*^2^ = 89%), including 544 individuals. The results of sensitivity analyses showed a good robustness of the association ([Supplementary-material supplementary-material-1]). Heterogeneity could be partly explained by the subgroup analysis. There was no difference of statistical significance in each subgroup (*P* = 0.21, *P* = 0.08, *P* = 0.45, *P* = 0.52) but not in patients administrated with probiotics except *Lactobacillus* spp. and *Bifidobacterium* spp. (*P* = 0.02), as shown in Figure 5(b).

#### 3.4.3. HOMA-IR

**Figure 6:**
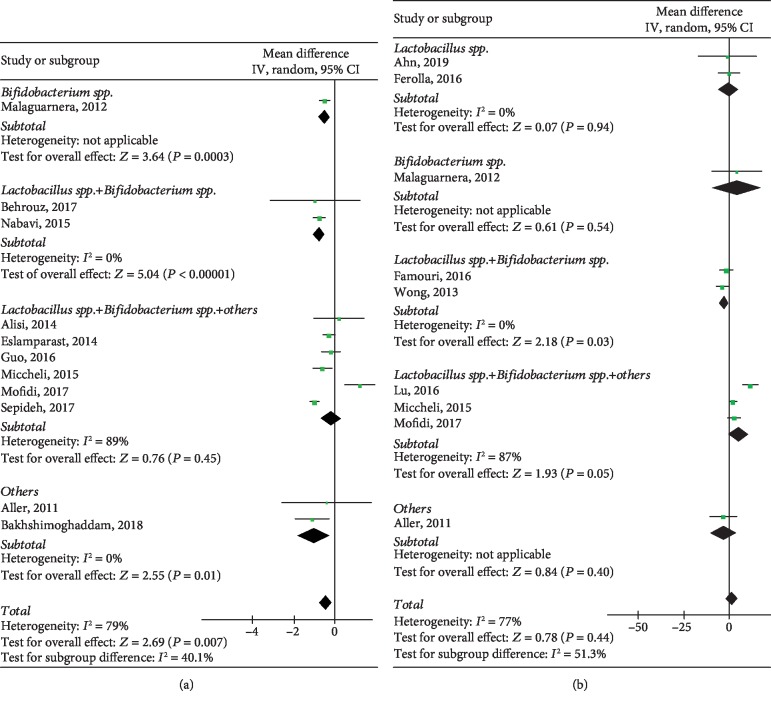
Forest plots of comparison for the effects of probiotics in NAFLD patients, showing (a) homeostasis model assessment-insulin resistance (HOMA-IR) and (b) high-density lipoprotein cholesterol (HDL-C).

Data pertinent to HOMA-IR were extracted from 11 heterogeneous studies (*P* < 0.00001, *I*^2^ = 79%) and included 569 individuals. Our meta-analysis results suggested a significant association between HOMA-IR and probiotics (WMD: -0.42, 95% CI: [-0.73, -0.12], *P* = 0.007). Sensitivity analyses showed a good robustness ([Supplementary-material supplementary-material-1]). Studies in *Lactobacillus* spp.+*Bifidobacterium* spp.+others subgroup still showed heterogeneity (*P* = 0.00001, *I*^2^ = 89%) after the subgroup analysis. There were significant differences in all subgroups (*P* = 0.0003, *P* < 0.00001, *P* < 0.00001, *P* = 0.01), as shown in Figure 6(a).

### 3.5. Impact of Probiotics on Lipid Profiles in NAFLD Patients

#### 3.5.1. HDL-C

Data regarding HDL-C extracted from 8 studies included 408 individuals, and the analyses found no significant association between the interventional group and the control group (WMD: 1.32, 95% CI: [-2.00, 4.64], *P* = 0.44) with obvious heterogeneity (*P* < 0.0001, *I*^2^ = 74%).  Figure 6(b) demonstrates that there was no difference in statistical significance in *Lactobacillus* spp. subgroup (*P* = 0.94), *Bifidobacterium* spp. subgroup (*P* = 0.54), and other probiotic subgroups (*P* = 0.40) while *Lactobacillus* spp.+*Bifidobacterium*spp. subgroup and *Lactobacillus* spp.+*Bifidobacterium* spp.+others subgroup showed a statistical significance (*P* = 0.03, *P* < 0.0001). Heterogeneity remained significant in *Lactobacillus* spp.+*Bifidobacterium* spp.+others subgroup (*P* < 0.0005, *I*^2^ = 87%), but not to the other subgroups.

#### 3.5.2. LDL-C

**Figure 7:**
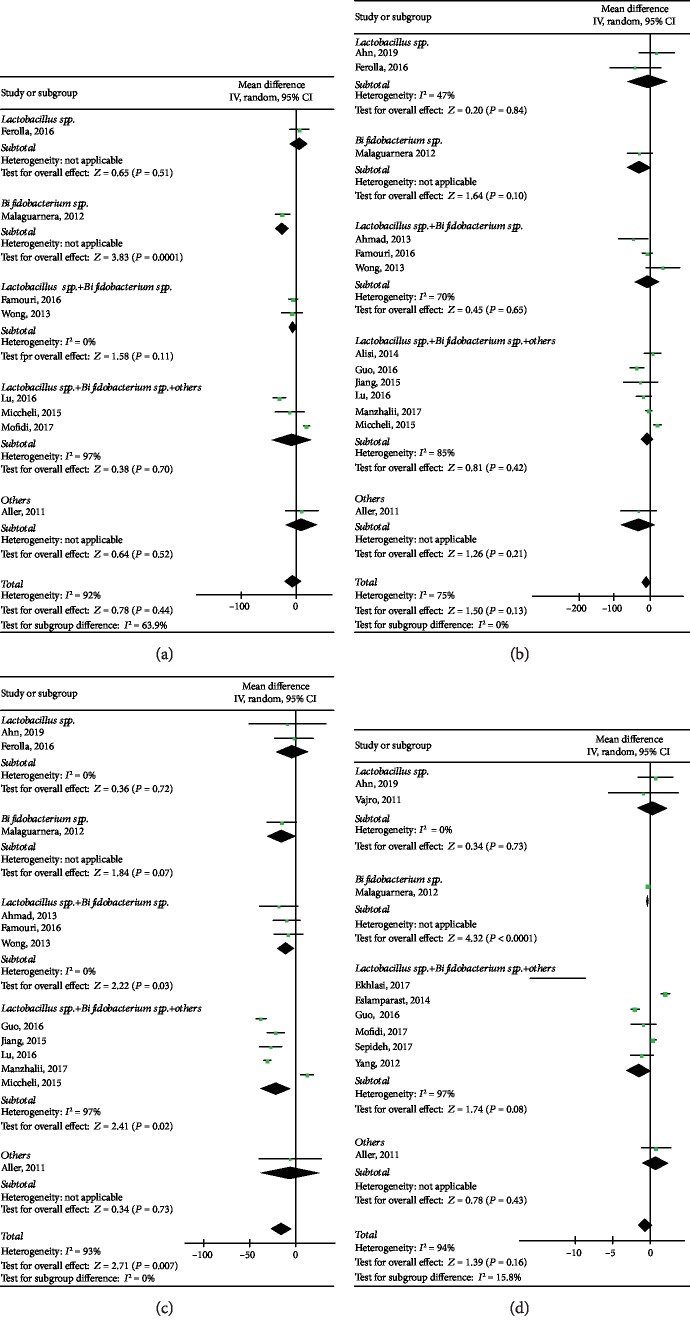
Forest plots of comparison for the effects of probiotics in NAFLD patients, showing (a) low-density lipoprotein cholesterol (LDL-C), (b) triglycerides (TG), (c) total cholesterol (TC), and (d) tumor necrosis factor-alpha (*Tnf-α*).

Results showed that there was no significant difference between the interventional group and the control group for LDL-C (WMD: -6.14, 95% CI: [-21.85, 9.30], *P* = 0.44) in eight heterogeneous studies (*P* < 0.00001, *I*^2^ = 92%), including 420 individuals. After subgroup analysis, the heterogeneity remained significant in *Lactobacillus* spp.+*Bifidobacterium* spp.+others subgroup (*P* < 0.00001). However, there were significant differences in statistical significance in *Bifidobacterium* spp. subgroup (*P* = 0.0001) and *Lactobacillus*spp.+*Bifidobacterium* spp.+others subgroup (*P* = 0.01), as shown in Figure 7(a).

#### 3.5.3. TG

In the case of TG, there was no statistic difference between the probiotics and the control group (WMD: -9.60, 95% CI: [-22.13, 2.93], *P* = 0.13) in 13 heterogeneous studies (*P* < 0.00001, *I*^2^ = 75%), including 766 individuals. The trim and filling computation suggested that it had no significant influence on the conclusion with no publication bias (*P* = 0.233) ([Supplementary-material supplementary-material-1]). Likewise, the results of sensitivity analyses showed a good robustness of the association. The subgroup analyses were unable to explain the heterogeneity in *Lactobacillus* spp.+*Bifidobacterium* spp. subgroup (*P* = 0.04) and *Lactobacillus* spp.+*Bifidobacterium* spp.+others subgroup (*P* < 0.00001). No significant difference was found in each subgroup (*P* = 0.96, *P* = 0.10, *P* = 0.18, *P* = 0.84, *P* = 0.21), as shown in Figure 7(b).

#### 3.5.4. TC

12 studies reported on TC between the interventional group and the control group. Results showed that TC was not significantly associated with probiotics (WMD: -15.38, 95% CI: [-26.50, -4.25], *P* = 0.007) with obvious heterogeneity (*P* < 0.00001, *I*^2^ = 93%), including 722 individuals. The results of sensitivity analyses demonstrated a good robustness of the association, without evidence of publication bias (*P* = 0.175) ([Supplementary-material supplementary-material-1]). The subgroup analysis failed to explain the source of heterogeneity in *Lactobacillus* spp.+*Bifidobacterium* spp.+others subgroup (*P* < 0.00001). However, there were significant differences in *Lactobacillus* spp.+*Bifidobacterium* spp. subgroup (*P* = 0.03) and *Lactobacillus* spp.+*Bifidobacterium* spp.+others subgroup (*P* = 0.02), but not in other subgroups (*P* = 0.72, *P* = 0.07, *P* = 0.73), as shown in Figure 7(c).

### 3.6. Impact of Probiotics on Inflammation Factors in NAFLD Patients

*Tnf-α* is considered to reflect inflammatory state. No significant correlation existed between *Tnf-α* and probiotics (WMD: -0.65, 95% CI: [-1.56, 0.27], *P* = 0.16) in 10 heterogeneous studies (*P* < 0.00001, *I*^2^ = 94%), including 479 individuals. The subgroup analysis could not well explain the source of heterogeneity, and the results showed that *Bifidobacterium* spp. subgroup has significant differences between probiotic and control individuals (*P* < 0.0001) (Figure 7(d)). Although there was no publication bias (*P* = 0.740), results of sensitivity analyses showed that the robustness of the association between *Tnf-α* and probiotics was not good, while there was a reversed conclusion after we exclude the item performed by Eslamparast et al. [27] (WMD: -1.00, 95% CI: [-1.87, -0.12], *P* = 0.03) ([Supplementary-material supplementary-material-1]). Besides, the trim and filling computation results also suggested a reversed result (from *P* = 0.165 to *P* = 0.004) ([Supplementary-material supplementary-material-1]).

### 3.7. Meta-Regression Analyses

For meta-regression analyses, several variables (population, region, duration, and lifestyle change) were eligible for inclusion in the univariable regression analysis. As shown in Table 3, all of the variables except population for BMI showed no influence on the effect of probiotics (*P* > 0.05) in NAFLD patients.

### 3.8. Quality of Evidence

The results of evidence quality assessment are shown in Supplemental [Supplementary-material supplementary-material-1]. For the outcome of BMI, the effect of probiotics was supported by moderate-quality evidence. For the outcome of liver function, the effects of probiotics were supported by high-quality evidence in ALT, moderate-quality evidence in AST, and low-quality evidence in GGT. For the outcome of glycemic indices, the effects of probiotics were supported by at least moderate-quality evidence in all indications. For the outcome of TC and TG, the effects of probiotics were supported by moderate-quality evidence. For the outcome of HDL-C and LDL-C, the effects of probiotics were supported by low-quality evidence. Inflammation factor was supported by low quality or very low quality of evidence.

### 3.9. Adverse Event

Few minor adverse events were reported: one patient complained of moderate headaches and two of musculoskeletal pain; two patients appeared dyspepsia, and both of which were resolved without reoccurrence. No serious adverse event was reported in all studies. We think there was no evidence to suggest that adverse events occurred are associated with probiotics untaken.

## 4. Discussion

Although a number of RCTs designed to identify efficacy and secure therapy for NAFLD are in progress [45–47], no agent has received approval by the Food and Drug Administration for the treatment of NAFLD as yet. Thus, it is necessary to update a systematic review to assess the efficacy of probiotics in NAFLD treatment. In this meta-analysis, we summarized evidence from 28 studies involving 1555 patients with NAFLD to assess the efficacies of probiotic interventions for several important outcomes, including BMI, liver functions, glycemic indices, lipid profiles, and inflammation factors. Overall, probiotics may play a more inspiring effect than we have ever predicted.

A recent analysis involving more than 8.5 million persons over 22 countries showed that 80% of patients with NAFLD are overweight or obese [4]. This information supports the concept that NAFLD is a metabolic syndrome with systemic disorder of energy homeostasis that accompanies hepatic adiposity [48]. Likewise, our results showed significant association between probiotics and BMI (*P* = 0.003) with a good stability, regardless of lifestyle intervention ([Supplementary-material supplementary-material-1]). Similar results have been confirmed by Gao et al. [49], but our meta-regression results suggested that age was one of the sources of heterogeneity because probiotics was not significantly associated with BMI in children subgroup (*P* = 0.27). The role of probiotics in obese children has been controversial for a long time. Trace back to 2008, Chouraqui et al. [50] indicated that infant formulas containing mixtures of probiotics had no significant effect on body weight changes on infants compared with the control group, but Alisi et al. [20] suggested probiotics could improve fat metabolism in obese children and contribute to weight loss. Recently, a different result was revealed in an age-based meta-analysis, which showed that probiotics could cause weight gain in children [51]. We considered a moderate grade of evidence on conclusions above due to the insufficient quality of included studies and high unexplained heterogeneity. Likewise, the insufficient number of children studies (four items) and the large heterogeneity (97%) are problems cannot be solved in our study as well. Therefore, the effect of probiotics in BMI of children with NAFLD is still unclear. The 2018 TES Obesity Management Science Statement did not recommend probiotics to treat obesity, which may result from the strain specific actions of probiotics and varies individual response in BMI. Above all, the role of probiotics in reducing BMI in adult patients with NAFLD is unequivocal, but more clinical evidences are needed in children. The reason for the different effects of probiotics among child and adult NAFLD patients should be explored in further studies. Besides, types and doses of probiotics may be key issues to be considered in NAFLD treatment.

NAFLD usually first suspected when a moderately increase was detected among ALT, AST, and GGT by liver function tests [4]. Our meta-analysis results showed that probiotics had a mitigating effect on ALT, AST, and GGT in patients with NAFLD. The subgroup analyses suggested a small dose of probiotics could still exert a protective effect on liver ([Supplementary-material supplementary-material-1]). Results of LSM demonstrated that probiotics could not reverse liver stiffness or liver histology; however, hepatic steatosis change defined by liver ultrasound showed a positive result ([Supplementary-material supplementary-material-1]). Our results are also supported by previous publications [52, 53]. Although Gao et al. [49] suggested that there exist some confusions on the effects of probiotics in improving liver functions due to the high heterogeneity and a lack of liver biopsy in their meta-analysis, our results showed a good robustness of association between probiotics and liver functions of NAFLD patients, which is difficult to get a opposite conclusion, regardless of the heterogeneity. Moreover, probiotics are also beneficial to abnormal liver functions caused by cirrhosis and alcoholic liver disease [54, 55]. The protective effect may due to an inhabitation of intestinal bacterial overgrowth and a reduction in serum endotoxin levels. In all, we believe that probiotics could improve liver functions (not limited to NAFLD) but seems no help to reverse the liver fibrosis. To consummate our conclusion, liver biopsy is needed in further researches.

It has been proved that hyperinsulinemia and insulin resistance (IR) are closely associated with NAFLD, and IR has strongly negative effects in liver metabolism [56]. Our meta-analysis results suggested a beneficial effect of probiotics in insulin level and IR but nonsignificant decrease in FBS. This notion is consistent with a recent review suggesting that probiotic supplementation may have a moderately beneficial effect on HOMA-IR control [52]. It is noteworthy that the results of FBS could be influenced by several factors, including patients' condition and test method. Different approaches between studies may lead to the instability of the FBS results and contribute to the instability. Altogether, although probiotics might not have direct impact on blood glucose level, they could contribute to insulin resistance improvement in NAFLD patients according to our study. The administration of probiotics appears to have a beneficial role in the management of glucose homeostasis in NAFLD patients. Furthermore, a unified standard for FBS measuring should be set up and strictly enforced by future related researches.

High liver fat content leads to increased serum fatty acids, but our results suggested a negative association between probiotics and lipid profiles. After the subgroup analysis by probiotic strains, effects of probiotics on the levels of HDL-C, LDL-C, and TC were only detected in few certain conditions. A previous meta-analysis of 30 clinical trials conducted by Cho and Kim [57] reported that there was no significant effect of probiotics on HDL-C or TG, while the effects of probiotics on TC and LDL-C depended on variety of factors, for example, baseline of TC level, treatment durations, and certain probiotic strains, which was also confirmed by Shimizu et al. [58]. The superiority of above meta-analyses is that they included more RCTs and performed more particular analyses, which is convincing. However, populations included in these meta-analyses were not restrict to NAFLD patients which might lead to confusion. Although there are theories for the effect of probiotics on regulating lipid profiles [59–61], a strong evidence on the effect of probiotics in NAFLD patients is absence currently. Studies included in our meta-analysis are still heterogeneous, although we had performed the subgroup analysis and meta-regression. We reserved about the effects of probiotics on the regulation of lipid profiles in NAFLD patients and suggest that they may not as effective as reported before. A range of confounding factors (region, baseline of TC level, treatment durations, and certain probiotic strains) which could disturb the results of lipid profiles were credited and should be taken notice in further clinical trials.

Some researchers proposed a “three-hit” theory to explain the development of NAFLD, including steatosis, lipotoxicity, and inflammation [62]. Steatosis results in increased signaling of NF-*κβ* and promotes a production of proinflammatory mediators like *Tnf-α*, which contribute to the recruitment and activation of Kupffer cells to mediate inflammation in NAFLD [63–65]. Our results suggested that probiotics had no significant efficacy in inflammation factors, but the sensitivity analysis results showed a reversed conclusion when we excluded the study by Eslamparast et al. [27]. After we reread the item, we think it is a high-quality RCT and cannot be excluded, which demonstrated that our conclusion on *Tnf-α* was instable. Little systematic review or meta-analysis has been performed regarding the role of probiotics in inflammation factors in NAFLD patients. Zarrati et al. [66] suggested that the expression of *Tnf-α* did not change with the use of probiotic yogurt. But Sepideh et al. [40] gave a significant result. Meta-analysis by Gao et al. [49] included four homogeneous studies indicated that probiotics had a positive effect in reducing *Tnf-α* levels in NAFLD patients, which may be a reference. In all, we think there is no strong evidence to confirm the effect of probiotics on inflammation factors, while more clinical trials are needed.

It should be noticed that the diversity of probiotic intervention employed by the different studies may result in confounding, which is important because lots of publications have proved that different species of probiotics may promote opposing effects in human beings [67]. However, complex existing data including doses, durations, pharmaceutical formulations, and combination of treatment differed in each study are difficult to reconcile. Still, we found that probiotics utilized in the included studies overlapped significantly, which were mainly characteristics by *Lactobacillus* or *Bifidobacterium* strains, or their combinations. To explore the effect of different probiotic strains, we conducted the subgroup analyses according to different probiotic formulations utilized (Figures 4–7). Interestingly, we found that *Bifidobacterium* spp. seems to perform a better effect than *Lactobacillus* spp. However, giving the fact that standardization in the form and course of currently marketed probiotic supplements is absence, it is difficult to perform direct comparisons between these formulations. But on the other hand, we think it is reasonable to accept the biological plausibility of probiotics for their positive effects according to previous clinical trials. In total, our results could not reach yield specific insights for the formulations or duration in the utilized of probiotics in NAFLD treatments. It is important and meaningful to obtain more in-depth comprehension in the role of gut microbiota in the pathogenesis of NAFLD, which may contribute to a recognized probiotic formulation and application method or achieve the more attractive individualized treatment.

No serious adverse event related to the administration of probiotics was found in this review. However, the trials included in this review excluded NAFLD patients with underlying conditions such as hepatitis B, hepatitis C, autoimmune hepatitis, liver decompensation, and genetic liver disease so that the side effect of probiotics in NAFLD patients with above diseases is unknown.

Limitations of our review, which are inherent to the nature of the individual studies and meta-analysis, need to be mentioned as well. (1) There was high unexplained heterogeneity among studies. To tackle this issue, a random effects model and sensitive analysis were applied to minimize the disturb of heterogeneity. Furthermore, the subgroup analysis and meta-regression were performed to find potential sources of heterogeneity. (2) Regardless of positive findings above, all the endpoints in these studies are surrogate outcome, not a hard endpoint (e.g., mortality). Considering the fact that it is impractical to perform large and long clinical trials to identify the treatment-related clinical benefits of probiotics due to the slow nature progressive of NAFLD, it is logical to assume the reduction of surrogate markers translates into reduction of cirrhosis, or liver-related mortality, while liver biopsy offers the best surrogate measure. But little study in our meta-analysis performed a histological feature because of the invasive in liver biopsy, which decreases the quality of evidence. (3) Literatures published in languages except English and Chinese were not detected, which result in selection bias.

BMI, ALT, AST, glycemic indices, TG, and TC showed at least moderate-quality evidence, while HDL-C, LDL-C, and *Tnf*-*α* suggested low or very low-quality evidence, which is mainly based on the small quantity of individuals included and high heterogeneity. To increase the quality of the summarized evidence, we strongly recommend that further clinical trials should pay more attention on indexes of liver fibrosis and inflammation factors. Despite limitations, this review provides an in-depth assessment of the effect of probiotics in NAFLD patients. As a final observation, since probiotics is affordable, widely available, and safe, we encourage NAFLD patients with obesity, abnormality liver enzymology, or hyperglycemia to use probiotics as a complementary therapeutic approach.

In conclusion, our meta-analysis clearly identifies probiotics as a common complementary therapeutic approach in NAFLD patients, which warrants attention. We clarified that probiotics is superior to placebo in improving BMI, liver enzymology, and hyperglycemia in NAFLD patients. Furthermore, more RCTs, particularly investigate indexes of liver fibrosis and inflammation factors, are warranted to further establish a more comprehensive assessment on the efficacy of probiotics in NAFLD patients, which would inform the development of relative practice guidelines in the future.

## Figures and Tables

**Table 1:** PICOS criteria for inclusion and exclusion of studies.

Parameter	Defined criteria for current study
P (population)	Patients with NAFLD
I (intervention)	Probiotic supplementation
C (comparison)	Placebo (product without microorganisms)
O (outcomes)	Effects of probiotic supplementation (body mass index, liver functions, blood glucose, blood lipids, inflammation index)
S (study design)	Randomized clinical trials

**Table 2:** Characteristics of the included studies.

Author, year	Region, period	Study design	Total = 1105	Mean age (y), male (%)	Intervention, *N* = ITT	Control, *N* = ITT	Diet/exercise by guide	Follow-up duration (w)
Abdel, 2017	Egypt, 2014-2016	NA, SC, PC	30	44, 56.67	*Lactobacillus acidophilus*, *N* = 15	Placebo, *N* = 15	NA	4
Shavakhi et al., 2013	Iran, 2010-2012	DB, SC, PC	63	40.1 ± 12.3, 50.80	*Lactobacillus acidophilus*, *Lactobacillus casei*, *Lactobacillus rhamnosus*, *Lactobacillus bulgaricus*, *Bifidobacterium breve*, *Bifidobacterium longum*, *N* = 31	Placebo, *N* = 32	Yes	24
Ahn et al., 2019	Korea, NA	DB, SC, PC	48	43.32 ± 12.9, 48.2	*L. acidophilus* CBT LA1, *L. rhamnosus* CBT LR5, *L. paracasei* CBT LPC5, *P. pentosaceus* CBT SL4, *B. lactis* CBT BL3, *B. breve* CBT BR3, *N* = 30	Placebo, *N* = 35	Yes	12
Alisi et al., 2014	Italy, 2012-2013	DB, SC, PC	44	10.5 (children), 54.55	VSL#3, *N* = 22	Placebo, *N* = 22	Yes	16
Aller et al., 2011	Spain, NA	DB, SC, PC	28	46.9 ± 13, 71.43	*Lactobacillus bulgaricus*, *Streptococcus thermophilus*, *N* = 14	Placebo, *N* = 14	NA	12
Asgharian et al., 2016	Iran, 2014-2014	DB, SC, PC	74	47.18 ± 1.7, 25.68	*Lactobacillus casei*, *Lactobacillus acidophilus*, *Lactobacillus rhamnosus*, *Lactobacillus bulgaricus*, *Bifidobacteriumbreve*, *Bifidobacterium longum*, *Streptococcus thermophilus*, *N* = 38	Placebo, *N* = 36	Yes	8
Bakhshimoghaddam et al., 2018	Iran, 2016-2017	DB, SC, PC	68	40 ± 8.7, 50	*Streptococcus thermophilus*, *Lactobacillus delbrueckii* subsp. *Bulgaricus*, *N* = 30	Placebo, *N* = 28	Yes	24
Behrouz et al., 2017	Iran, 2015	DB, SC, PC	60	38.45 ± 8.6, 71.7	*Lactobacillus casei*, *Lactobacillus rhamnosus*, *Lactobacillus acidophilus*, *Bifidobacterium longum*, *Bifidobacterium breve*, *N* = 30	Placebo, *N* = 30	Yes	12
Cakir et al., 2017	Turkey, NA	DB, SC, PC	60	12.2 ± 2.1 (children), 66.7	*Bifidobacterium lactis*, *Lactobacillus acidophilus*, *Lactobacillus casei*, *N* = 28	Placebo, *N* = 30	Yes	16
Ekhlasi et al., 2016	Iran, 2012-2013	DB, SC, PC	30	42.5, NA	*Lactobacillus casei*, *Lactobacillus rhamnosus*, *Streptococcus thermophilus*, *Bifidobacterium breve*, *Lactobacillus acidophilus*, *Bifidobacterium longum*, *Lactobacillus bulgaricus*, *N* = 15	Placebo, *N* = 15	NA	8
Eslamparast et al., 2014	Iran, 2012-2012	DB, MC, PC	46	46 ± 9.2, 48.08	*Lactobacillus casei*, *Lactobacillus rhamnosus*, *Streptococcus thermophilus*, *Bifidobacterium breve*, *Lactobacillus acidophilus*, *Bifidobacterium longum*, *Lactobacillus bulgaricus*, *N* = 24	Placebo, *N* = 22	Yes	28
Famouri et al., 2017	Iran, 2014-2014	DB, SC, PC	64	12.65 ± 1.95 (children), 50	*Lactobacillus acidophilus*, *Bifidobacterium lactis*, *B. bifidu m*, *L. rhamnosus*, *N* = 32	Placebo, *N* = 32	Yes	12
Ferolla et al., 2016	Brazil, 2014-2015	NA, SC, PC	50	57.3, 24	Synbiotic, *L. reuteri*, *N* = 26	Placebo, *N* = 23	Yes	12
Guo et al., 2016	China, 2011-2013	NA, SC, PC	84	50.1 ± 12.1, 58.33	*Bifidobacterium longum*, *Lactobacillus acidophilus*, *Enterococcus faecalis*, *N* = 40	Placebo, *N* = 40	Yes	8
Javadi et al., 2017	Iran, 2013-2014	DB, SC, PC	39	42 ± 8.9, 76.92	*Bifidobacterium longum*, *Lactobacillus acidophilus*, *N* = 20	Placebo, *N* = 19	NA	12
Jiang et al., 2015	China, 2014-2015	NA, SC, PC	62	42.58, 53.03	*Bifidobacterium longum*, *Lactobacillus acidophilus*, *Enterococcus faecalis*, *N* = 31	Placebo, *N* = 30	Yes	12
Kobyliak et al., 2018	Ukraine, NA	DB, SC, PC	58	55.3 ± 10, NA	*Lactobacillus*, *Lactococcus*, *Bifidobacterium*, *Propionibacterium*, *Acetobacter*, *N* = 30	Placebo, *N* = 28	Yes	8
Lu et al., 2016	China, 2014-2015	NA, SC, PC	120	45.76 ± 6.66, 68.30	*Bifidobacterium infantis*, *Lactobacillus acidophilus*, *Enterococcus faecalis*, *Bacillus cereus*, *N* = 60	Placebo, *N* = 60	Yes	4
Malaguarnera et al., 2012	Italy, 2003-2006	DB, SC, PC	66	46.8 ± 5.55, 48.48	*Bifidobacterium longum*, *N* = 34	Placebo, *N* = 32	Yes	24
Manzhalii et al., 2017	Ukraine, NA	NA, SC, PC	66	43.7 ± 1.4, 56	*Lactobacillus casei*, *L. rhamnosus*, *L. bulgaris*, *Bifidobacterium longum*, *Streptococcus thermophilus*, *N* = 38	Placebo, *N* = 37	Yes	12
Miccheli et al., 2015	Italy, NA	DB, SC, PC	31	10.5 (children), 54.84	VSL#3, *N* = 15	Placebo, *N* = 16	Yes	16
Mofidi et al., 2017	Iran, NA	DB, SC, PC	42	42.35 ± 10.78, 54.76	*Lactobacillus casei*, *Lactobacillus rhamnosus*, *Streptococcus thermophilus*, *Bifidobacterium breve*, *Lactobacillus acidophilus*, *Bifidobacterium longum*, *Lactobacillus bulgaricus*, *N* = 21	Placebo, *N* = 21	Yes	28
Nabavi et al., 2015	Iran, NA	DB, SC, PC	72	43.4 ± 7.93, 48.61	Probiotic yogurts (*Lactobacillus bulgaricus*, *Streptococcus thermophilus*, *B. lactis Bb12*, *L. acidophilus La5*) *N* = 36	Conventional yogurts, *N* = 36	NA	8
Sepideh et al., 2016	Iran, 2013-2013	DB, SC, PC	42	44.7 ± 2.26, 66.67	*Lactobacillus casei*, *Lactobacillus acidophilus*, *Lactobacillus rhamnosus*, *Lactobacillus bulgaricus*, *Bifidobacterium breve*, *Bifidobacterium longum*, *Streptococcus thermophilus*, *N* = 21	Placebo, *N* = 21	NA	8
Vajro et al., 2011	Italy, NA	DB, SC, PC	20	10.7 ± 2.1 (children), 90	*Lactobacillus GG*, *N* = 10	Placebo, *N* = 10	NA	8
Wong et al., 2013	China, 2009-2009	DB, SC, PC	20	48.5 ± 9, 65	*Lactobacillus plantarum*, *Lactobacillus delbrueckii ssp*. *bulgaricus*, *Lactobacillus acidophilus*, *Lactobacillus rhamnosus*, *Bifidobacterium bifidum*, *N* = 10	Placebo, *N* = 10	Yes	24
Yang et al., 2012	China, 2010-2011	NA, SC, PC	60	47.5 ± 12.3, 53.3	*Bacillus subtilis*, *Enterococcus*, *N* = 30	Placebo, *N* = 30	Yes	4
Yao et al., 2013	China, 2010-2012	DB, SC, PC	108	45.75 ± 11.8, 58.30	*Bifidobacterium longum*, *Lactobacillus acidophilus*, *Enterococcus faecalis*, *N* = 55	Placebo, *N* = 53	Yes	12

DB: double blinded, SC: single center, MC: multicenter, PC: placebo controlled, NA: not available, ITT: intention to treat.

**Table 3:** Univariable predictors with meta-regression on the effect of probiotics.

	Variable	No	Regression coefficient (95% CI)	SE	*P* value
BMI					
Population	Adults	13	1	—	—
	Children	2	-0.3 (-0.1 to -0.6)	0.12	0.013
ALT					
Region	Asia	13	1	—	—
	Europe	6	-4.6 (-6 to -1.4)	0.76	0.09
	US or others	1	-5.5 (-9.4 to -0.7)	2.15	0.31
AST					
Duration	4-12 w	11	1	—	—
	12-28 w	6	-3.5 (-9.6 to -0.8)	0.9	0.54
FBS					
Region	Asia	8	1	—	—
	Europe	4	2 (0.2 to 5.2)	0.63	0.62
	US or other	1	-3.5 (-5.6 to 1.1)	1.68	0.78
Insulin					
Duration	4-12 w	5	1	—	—
	12-28 w	5	-0.07 (-1.92 to -0.01)	0.21	0.11
HOMA-IR					
Lifestyle	Maintain original lifestyle	4	1	—	—
	Follow the guidelines	7	-0.03 (-0.04 to -0.01)	0.005	0.23
TG					
Region	Asia	8	1	—	—
	Europe	4	-7.8 (-11.1 to 1.2)	1.54	0.18
	US or others	1	1.1 (-2.1 to 5)	1.78	0.65
TC					
Duration	4-12 w	8	1	—	—
	12-28 w	4	-5.8 (-7.9 to -0.1)	0.98	0.52
*Tnf-α*					
Lifestyle	Maintain original lifestyle	4	1	—	—
	Follow the guidelines	6	-0.04 (-0.08 to -0.01)	0.007	0.44

SE: standard error, BMI: body mass index, ALT: alanine aminotransferase, AST: aspartate transaminase, FBS: fasting blood sugar, HOMA-IR: homeostasis model assessment-insulin resistance, TG: triglycerides, TC: total cholesterol, *Tnf-α*: tumor necrosis factor-alpha.

## Supplementary Materials

Supplementary Materials Electronic Supplementary Information (ESI) available. Supplemental Table 1: the checklist of PRISMA statement. Figure S1: more details on search strategy, supplementary data of body mass index. Figure S2: supplementary data of alanine aminotransferase. Figure S3: supplementary data of aspartate transaminase. Figure S4: supplementary data of fasting blood sugar. Figure S5: supplementary data of insulin. Figure S6: supplementary data of homeostasis model assessment-insulin resistance. Figure S7: supplementary data of triglycerides. Figure S8: supplementary data of total cholesterol. Figure S9: supplementary data of tumor necrosis factor. Supplemental Table 2: evidence quality assessment. Figure S10: forest plots of comparison for the effects of probiotics in NAFLD patients. This material is available free of charge via the Internet at DOI: x0xx00000x.
