# Supplementary material for: Systematic Review with Meta-Analysis: The Effects of Probiotics in Nonalcoholic Fatty Liver Disease
Source: Gastroenterol Res Pract. 2019 Dec 11;2019:1484598. doi: 10.1155/2019/1484598 (PMC6927028; doi:10.1155/2019/1484598)
Supplement: Supplementary Materials — Electronic Supplementary Information (ESI) available. Supplemental Table 1: the checklist of PRISMA statement. Figure S1: more details on search strategy, supplementary data of body mass index. Figure S2: supplementary data of alanine aminotransferase. Figure S3: supplementary data of aspartate transaminase. Figure S4: supplementary data of fasting blood sugar. Figure S5: supplementary data of insulin. Figure S6: supplementary data of homeostasis model assessment-insulin resistance. Figure S7: supplementary data of triglycerides. Figure S8: supplementary data of total cholesterol. Figure S9: supplementary data of tumor necrosis factor. Supplemental Table 2: evidence quality assessment. Figure S10: forest plots of comparison for the effects of probiotics in NAFLD patients. This material is available free of charge via the Internet at DOI: x0xx00000x. [file 1484598.f1.pdf]

SUPPORTING INFORMATION

Systematic review with meta-analysis: the effects of probiotics in non-alcoholic fatty liver disease

Meng-Wei Xiao[1,2], Shi-Xin Lin[3], Zhao-Hua Shen[1,2], Wei-Wei Luo[1,2], and Xiao-Yan Wang[1,2,*]

[1] Department of Gastroenterology, Third Xiangya Hospital, Central South University, Changsha 410013, China

[2] Hunan Key Laboratory of Nonresolving Inflammation and Cancer, Changsha 410013, China

[3] Department of gynecology and obstetrics, Third Affiliated Hospital of Nanchang University, Nanchang 330000, China.

Correspondence should be addressed to Xiao-Yan Wang;
wxy220011@163.com

Supplemental table 1: Checklist of items in reporting a systematic review or meta-analysis (PRISMA statement)

Section/topic	Item	Checklist item	Reported on page
**Title**			
Title	1	Identify the report as a systematic review, meta-analysis, or both	1
**Abstract**			
Structured summary	2	Provide a structured summary including, as applicable, background, objectives, data sources, study eligibility criteria, participants, interventions, study appraisal and synthesis methods, results, limitations, conclusions and implications of key findings, systematic review registration number	1-2
**Introduction**			
Rationale	3	Describe the rationale for the review in the context of what is already known	2
Objectives	4	Provide an explicit statement of questions being addressed with reference to participants, interventions, comparisons, outcomes, and study design (PICOS)	2
**Methods**			
Protocol and registration	5	Indicate if a review protocol exists, if and where it can be accessed (such as web address), and, if available, provide registration information including registration number	NA
Eligibility criteria	6	Specify study characteristics (such as PICOS, length of follow-up) and report characteristics (such as years considered, language, publication status) used as criteria for eligibility, giving rationale	3
Information sources	7	Describe all information sources (such as databases with dates of coverage, contact with study authors to identify additional studies) in the search and date last searched	3
Search	8	Present full electronic search strategy for at least one database, including any limits used, such that it could be repeated	Supplementary data
Study selection	9	State the process for selecting studies (that is, screening, eligibility, included in systematic review, and, if applicable, included in the meta-analysis)	3
Data collection process	10	Describe method of data extraction from reports (such as piloted forms, independently, in duplicate) and any processes for obtaining and confirming data from investigators	4
Data items	11	List and define all variables for which data were sought (such as PICOS, funding sources) and any assumptions and simplifications made	4
Risk of bias in individual studies	12	Describe methods used for assessing risk of bias of individual studies (including specification of whether this was done at the study or outcome level), and how this information is to be used in any data synthesis	4
Summary measures	13	State the principal summary measures (such as risk ratio, difference in means	4-5
Synthesis of results	14	Describe the methods of handling data and combining results of studies, if done, including measures of consistency (such as I2 statistic) for each meta-analysis	4-5
Risk of bias across studies	15	Specify any assessment of risk of bias that may affect the cumulative evidence (such as publication bias, selective reporting within studies)	4-5
Additional analyses	16	Describe methods of additional analyses (such as sensitivity or subgroup analyses, meta-regression), if done, indicating which were pre-specified	4-5
**Results**			
Study selection	17	Give numbers of studies screened, assessed for eligibility, and included in the review, with reasons for exclusions at each stage, ideally with a flow diagram	5-6, Fig 1
Study characteristics	18	For each study, present characteristics for which data were extracted (such as study size, PICOS, follow-up period) and provide the citations	Table1

Risk of bias within studies	19	Present data on risk of bias of each study and, if available, any outcome-level assessment (see item 12).	Supplementary data
Results of individual studies	20	For all outcomes considered (benefits or harms), present for each study (a) simple summary data for each intervention group and (b) effect estimates and confidence intervals, ideally with a forest plot	Fig 3
Synthesis of results	21	Present results of each meta-analysis done, including confidence intervals and measures of consistency	5-21
Risk of bias across studies	22	Present results of any assessment of risk of bias across studies (see item 15)	Supplementary data
Additional analysis	23	Give results of additional analyses, if done (such as sensitivity or subgroup analyses, meta-regression) (see item 16)	5-21
**Discussion**			
Summary of evidence	24	Summarise the main findings including the strength of evidence for each main outcome; consider their relevance to key groups (such as health care providers, users, and policy makers)	22-25
Limitations	25	Discuss limitations at study and outcome level (such as risk of bias), and at review level (such as incomplete retrieval of identified research, reporting bias)	25
Conclusions	26	Provide a general interpretation of the results in the context of other evidence, and implications for future research	26
**Funding**			
Funding	27	Describe sources of funding for the systematic review and other support (such as supply of data) and role of funders for the systematic review	26

SEARCH STRATEGY (till to 2019.5.6)

PubMed

Query:

((((((((probiotic*[Title/Abstract]) OR prebiotic*[Title/Abstract]) OR synbiocit*[Title/Abstract]) OR bifidobacter*[Title/Abstract]) OR Lactobacill*[Title/Abstract]) OR flora[Title/Abstract])) OR "Probiotics"[Mesh])) AND (((((((((((((Non alcoholic Fatty Liver Disease[Title/Abstract]) OR NAFLD[Title/Abstract]) OR Nonalcoholic Fatty Liver Disease[Title/Abstract]) OR Fatty Liver, Nonalcoholic[Title/Abstract]) OR Fatty Livers, Nonalcoholic[Title/Abstract]) OR Liver, Nonalcoholic Fatty[Title/Abstract]) OR Livers, Nonalcoholic Fatty[Title/Abstract]) OR Nonalcoholic Fatty Liver[Title/Abstract]) OR Nonalcoholic Fatty Livers[Title/Abstract]) OR Nonalcoholic Steatohepatitis[Title/Abstract]) OR Nonalcoholic Steatohepatitides[Title/Abstract]) OR Steatohepatitides, Nonalcoholic[Title/Abstract]) OR Steatohepatitis, Nonalcoholic[Title/Abstract]) OR NASH[Title/Abstract] OR "Non-alcoholic Fatty Liver Disease"[Mesh])

Result: 358

Embase

Query:

('nonalcoholic fatty liver'/exp OR 'non alcoholic fatty liver disease':ab,ti OR 'nafld':ab,ti OR 'nonalcoholic fatty liver disease':ab,ti OR 'fatty liver, nonalcoholic':ab,ti OR 'fatty livers, nonalcoholic':ab,ti OR 'liver, nonalcoholic fatty':ab,ti OR 'livers, nonalcoholic fatty':ab,ti OR 'nonalcoholic fatty liver':ab,ti OR 'nonalcoholic fatty livers':ab,ti OR 'nonalcoholic steatohepatitis':ab,ti OR 'nonalcoholic steatohepatitides':ab,ti OR 'steatohepatitides, nonalcoholic':ab,ti OR 'steatohepatitis, nonalcoholic':ab,ti OR 'nash':ab,ti) AND ('probiotic agent'/exp OR 'probiotic*':ab,ti OR 'prebiotic*':ab,ti OR 'synbiocit*':ab,ti OR 'bifidobacter*':ab,ti OR 'lactobacill*':ab,ti OR 'flora':ab,ti)

Result: 842

Cochrane Library

Query:

D Search Hits
#1 MeSH descriptor: [Non-alcoholic Fatty Liver Disease] explode all trees 659
#2 Non alcoholic Fatty Liver Disease 1560
#3 NAFLD 1418
#4 Nonalcoholic Fatty Liver Disease 2014
#5 Fatty Liver, Nonalcoholic 2225
#6 Fatty Livers, Nonalcoholic 16
#7 Liver, Nonalcoholic Fatty 2225
#8 Livers, Nonalcoholic Fatty 16
#9 Nonalcoholic Fatty Liver 2225
#10 Nonalcoholic Fatty Livers16
#11 Nonalcoholic Steatohepatitis 892
#12 Nonalcoholic Steatohepatitides 0
#13 Steatohepatitides, Nonalcoholic 0
#14 Steatohepatitis, Nonalcoholic 892
#15 NASH 1545
#16 MeSH descriptor: [Probiotics] explode all trees 1776
#17 probiotic* 5727
#18 prebiotic* 1312
#19 synbiocit* 0
#20 bifidobacter* 2469
#21 Lactobacill* 4461
#22 flora 3838
#23 #1 or #2 or #3 or #4 or #5 or #6 or #7 or #8 or #9 or #10 or #11 or #12 or #13 or #14 or #15 3248
#24 #16 or #17 or #18 or #19 or #20 or #21 or #22 10806
#25 #23 and #24 128

Result: 128

Web of Science

Query:

TS= ((probiotic* OR prebiotic* OR synbiocit* OR bifidobacter* OR Lactobacill* OR flora) AND (Non alcoholic Fatty Liver Disease OR NAFLD OR Nonalcoholic Fatty Liver Disease OR Fatty Liver, Nonalcoholic OR Fatty Livers, Nonalcoholic OR Liver, Nonalcoholic Fatty OR Livers, Nonalcoholic Fatty OR Nonalcoholic Fatty Liver OR Nonalcoholic Fatty Livers OR Nonalcoholic Steatohepatitis OR Nonalcoholic Steatohepatitides OR Steatohepatitides, Nonalcoholic OR Steatohepatitis, Nonalcoholic OR NASH)) OR TI= ((probiotic* OR prebiotic* OR synbiocit* OR bifidobacter* OR Lactobacill* OR flora) AND (Non alcoholic Fatty Liver Disease OR NAFLD OR Nonalcoholic Fatty Liver Disease OR Fatty Liver, Nonalcoholic OR Fatty Livers, Nonalcoholic OR Liver, Nonalcoholic Fatty OR Livers, Nonalcoholic Fatty OR Nonalcoholic Fatty Liver OR Nonalcoholic Fatty Livers OR Nonalcoholic Steatohepatitis OR Nonalcoholic Steatohepatitides OR Steatohepatitides, Nonalcoholic OR Steatohepatitis, Nonalcoholic OR NASH))

Result: 752

OVID

Query:

(probiotic*.ab. or probiotic*.ti. or prebiotic*.ab. or prebiotic*.ti. or synbiocit*.ab. or synbiocit*.ti. or bifidobacter*.ab. or bifidobacter*.ti. or Lactobacill*.ab. or Lactobacill*.ti. or flora.ab. or flora.ti. or Probiotics.ab. or Probiotics.ti.) and (((((((((((((((((((((((((((((Non alcoholic Fatty Liver Disease.ab.) or Non alcoholic Fatty Liver Disease.ti.) or NAFLD.ab.) or NAFLD.ti.) or Nonalcoholic Fatty Liver Disease.ab.) or Nonalcoholic Fatty Liver Disease.ti.) or Fatty Liver, Nonalcoholic.ab.) or Fatty Liver, Nonalcoholic.ti.) or Fatty Livers, Nonalcoholic.ab.) or Fatty Livers, Nonalcoholic.ti.) or Liver, Nonalcoholic Fatty.ab.) or Liver, Nonalcoholic Fatty.ti.) or Livers, Nonalcoholic Fatty.ab.) or Livers, Nonalcoholic Fatty.ti.) or Nonalcoholic Fatty Liver.ab.) or Nonalcoholic Fatty Liver.ti.) or Nonalcoholic Fatty Livers.ab.) or Nonalcoholic Fatty Livers.ti.) or Nonalcoholic Steatohepatitis.ab.) or Nonalcoholic Steatohepatitis.ti.) or Nonalcoholic Steatohepatitides.ab.) or Nonalcoholic Steatohepatitides.ti.) or Steatohepatitides, Nonalcoholic.ab.) or Steatohepatitides, Nonalcoholic.ti.) or Steatohepatitis, Nonalcoholic.ab.) or Steatohepatitis, Nonalcoholic.ti.) or NASH.ab.) or NASH.ti.))

Result: 1026

SUPPLEMENTARY FIGURES

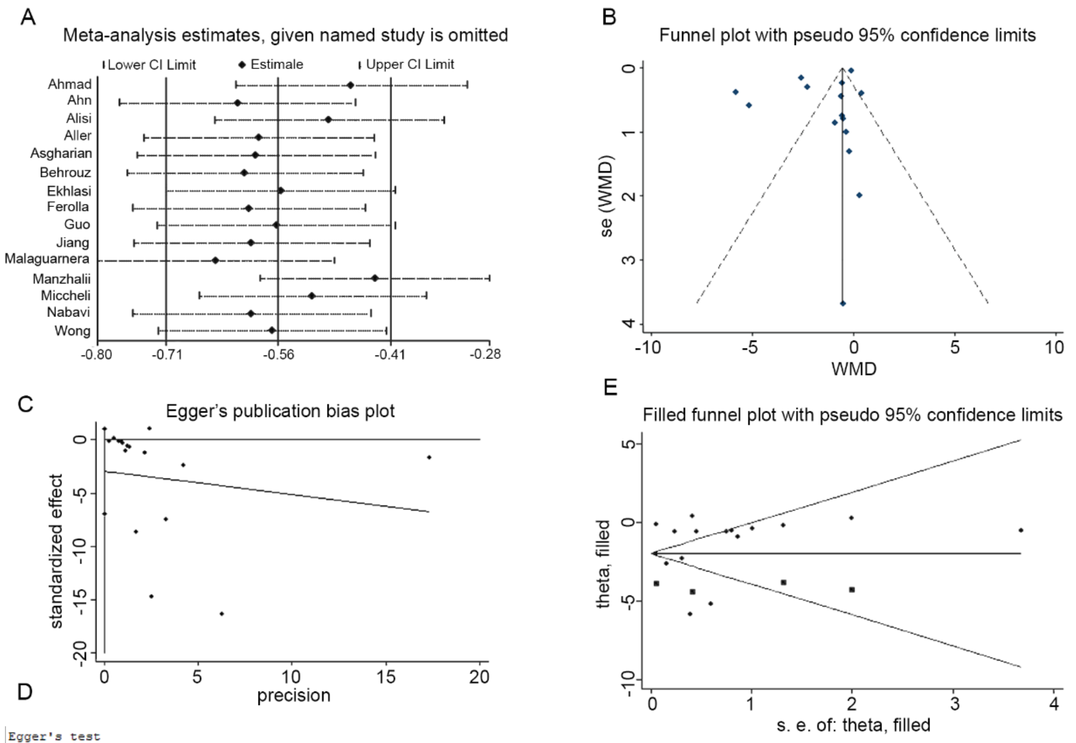

D

Egger's test

Std_Eff	Coef.	Std. Err.	t	P>|t|	[95% Conf.	Interval]
slope	-.2173143	.3567507	-0.61	0.553	-.9880273	.5533986
bias	-2.966082	1.828782	-1.62	0.129	-6.916927	.9847618

F

```
Meta-analysis

       |  Pooled      95% CI         Asymptotic      No. of
Method |     Est    Lower    Upper  z_value  p_value   studies
-------+----------------------------------------------------
Fixed  |  -0.565  -0.663  -0.466  -11.210    0.000      15
Random |  -1.460  -2.441  -0.480   -2.921    0.003

Test for heterogeneity: Q= 501.612 on 14 degrees of freedom (p= 0.000)
Moment-based estimate of between studies variance =  3.037

Trimming estimator: Linear
Meta-analysis type: Random-effects model

iteration |  estimate    Tn    # to trim     diff
----------+--------------------------------------
    1     |   -1.460     80        3          120
    2     |   -1.867     88        4           16
    3     |   -1.981     88        4            0

Filled
Meta-analysis

       |  Pooled      95% CI         Asymptotic      No. of
Method |     Est    Lower    Upper  z_value  p_value   studies
-------+----------------------------------------------------
Fixed  |  -2.012  -2.086  -1.938  -53.265    0.000      19
Random |  -1.952  -3.080  -0.825   -3.394    0.001

Test for heterogeneity: Q= 2389.318 on 18 degrees of freedom (p= 0.000)
Moment-based estimate of between studies variance =  5.375
```

Figure S1: Supplementary data of body mass index (BMI), showing the interpretation of single study (A), funnel plot (B), Egger's tests (C, D), trim-and-fill computation (E, F).

A

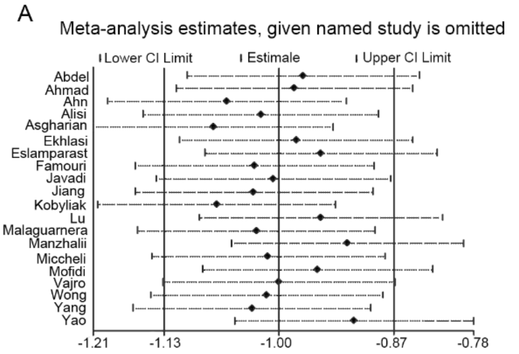

B

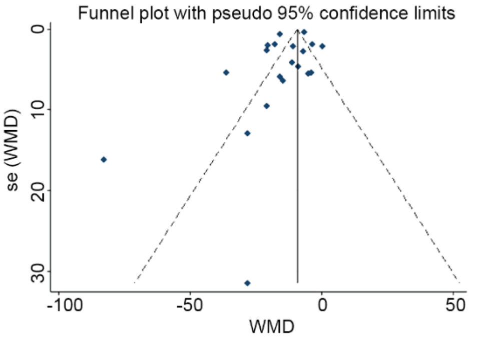

C

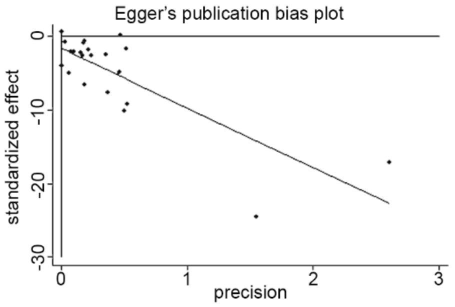

E

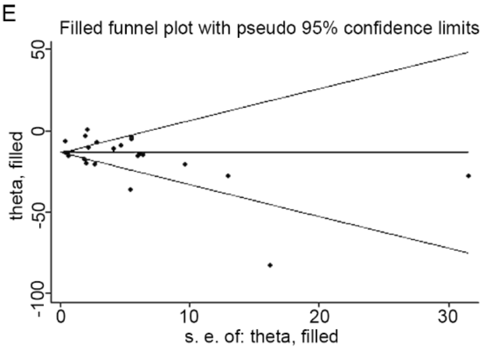

D

Egger's test

Std_Eff	Coef.	Std. Err.	t	P>|t|	[95% Conf. Interval]	
slope	-8.071722	1.455642	-5.55	0.000	-11.12991	-5.013531
bias	-1.690725	1.078887	-1.57	0.135	-3.957383	.5759325

F

```
Meta-analysis

       |  Pooled       95% CI         Asymptotic      No. of
Method |     Est    Lower   Upper  z_value  p_value   studies
-------+----------------------------------------------------
Fixed  |  -9.449 -10.041  -8.858  -31.321   0.000        20
Random | -13.401 -17.034  -9.769   -7.231   0.000

Test for heterogeneity: Q= 302.544 on 19 degrees of freedom (p= 0.000)
Moment-based estimate of between studies variance = 45.727

Trimming estimator: Linear
Meta-analysis type: Random-effects model

iteration |  estimate     Tn    # to trim     diff
----------+--------------------------------------
    1     |   -13.401     89        0          210
    2     |   -13.401     89        0            0

Note: no trimming performed; data unchanged

Filled
Meta-analysis

       |  Pooled       95% CI         Asymptotic      No. of
Method |     Est    Lower   Upper  z_value  p_value   studies
-------+----------------------------------------------------
Fixed  |  -9.449 -10.041  -8.858  -31.321   0.000        20
Random | -13.401 -17.034  -9.769   -7.231   0.000

Test for heterogeneity: Q= 302.544 on 19 degrees of freedom (p= 0.000)
Moment-based estimate of between studies variance = 45.727
```

Figure S2: Supplementary data of alanine aminotransferase (ALT), showing the interpretation of single study (A), funnel plot (B), Egger's tests (C, D), trim-and-fill computation (E, F).

A

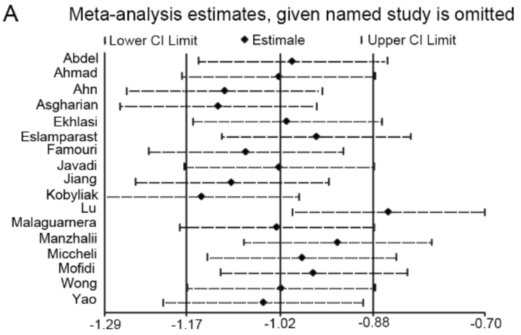

B

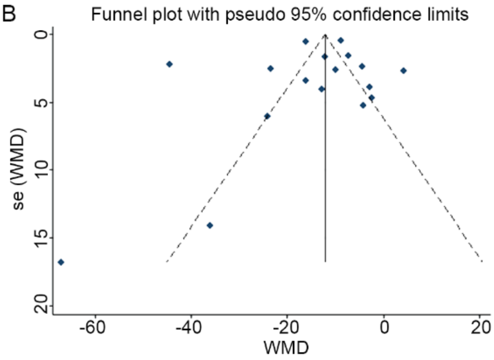

C

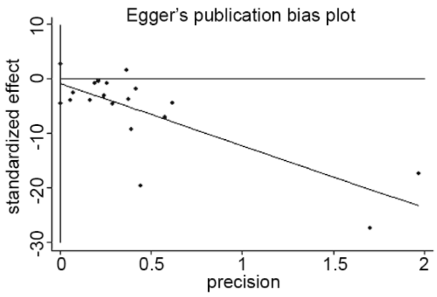

E

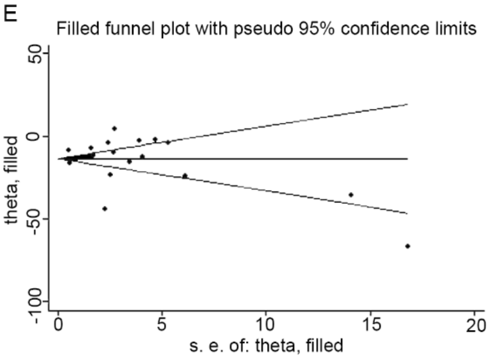

D

Egger's test

Std_Eff	Coef.	Std. Err.	t	P>|t|	[95% Conf.	Interval]
slope	-11.40297	2.405359	-4.74	0.000	-16.52987	-6.276069
bias	-.819056	1.712781	-0.48	0.639	-4.469761	2.831649

F

Meta-analysis

Method	Pooled Est	95% CI Lower	95% CI Upper	Asymptotic z_value	Asymptotic p_value	No. of studies
Fixed	-12.196	-12.864	-11.529	-35.807	0.000	17
Random	-13.539	-17.862	-9.217	-6.139	0.000	

Test for heterogeneity: Q= 398.324 on 16 degrees of freedom (p= 0.000)
Moment-based estimate of between studies variance = 65.353

Trimming estimator: Linear
Meta-analysis type: Random-effects model

iteration	estimate	Tn	# to trim	diff
1	-13.539	77	0	153
2	-13.539	77	0	0

Note: no trimming performed; data unchanged

Filled
Meta-analysis

Method	Pooled Est	95% CI Lower	95% CI Upper	Asymptotic z_value	Asymptotic p_value	No. of studies
Fixed	-12.196	-12.864	-11.529	-35.807	0.000	17
Random	-13.539	-17.862	-9.217	-6.139	0.000	

Test for heterogeneity: Q= 398.324 on 16 degrees of freedom (p= 0.000)
Moment-based estimate of between studies variance = 65.353

Figure S3: Supplementary data of aspartate transaminase (AST), showing the interpretation of single study (A), funnel plot (B), Egger's tests (C, D), trim-and-fill computation (E, F).

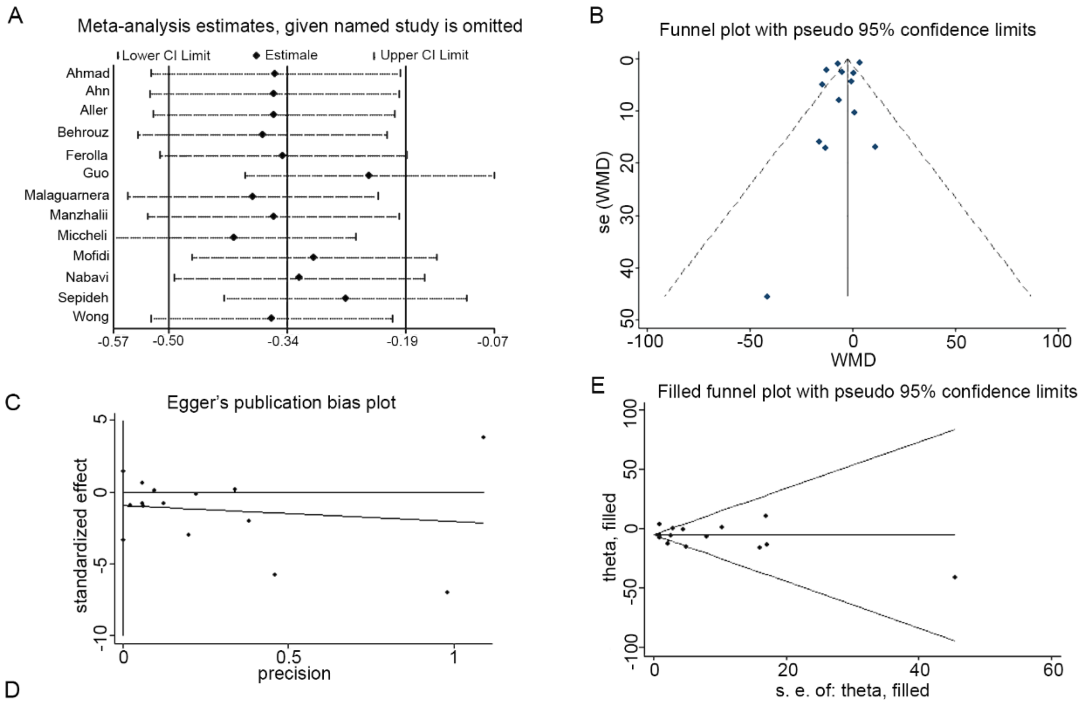

D

```
Egger's test

     Std_Eff |      Coef.   Std. Err.      t    P>|t|     [95% Conf. Interval]
-------------+----------------------------------------------------------------
       slope |  -1.131877   2.376196    -0.48   0.643    -6.361849    4.098095
        bias | -.9304704    1.092596    -0.85   0.413    -3.335259    1.474318
```

F

```
Meta-analysis

       |  Pooled      95% CI         Asymptotic      No. of
Method |     Est    Lower    Upper  z_value  p_value   studies
-------+----------------------------------------------------
Fixed  |  -2.518   -3.700   -1.336   -4.175   0.000      13
Random |  -4.983   -9.950   -0.016   -1.966   0.049

Test for heterogeneity: Q= 96.554 on 12 degrees of freedom (p= 0.000)
Moment-based estimate of between studies variance = 45.291

Trimming estimator: Linear
Meta-analysis type: Random-effects model

iteration |  estimate    Tn    # to trim     diff
----------+--------------------------------------
    1     |   -4.983     36        0           91
    2     |   -4.983     36        0            0

Note: no trimming performed; data unchanged

Filled
Meta-analysis

       |  Pooled      95% CI         Asymptotic      No. of
Method |     Est    Lower    Upper  z_value  p_value   studies
-------+----------------------------------------------------
Fixed  |  -2.518   -3.700   -1.336   -4.175   0.000      13
Random |  -4.983   -9.950   -0.016   -1.966   0.049

Test for heterogeneity: Q= 96.554 on 12 degrees of freedom (p= 0.000)
Moment-based estimate of between studies variance = 45.291
```

Figure S4: Supplementary data of fasting blood sugar (FBS), showing the interpretation of single study (A), funnel plot (B), Egger's tests (C, D), trim-and-fill computation (E, F).

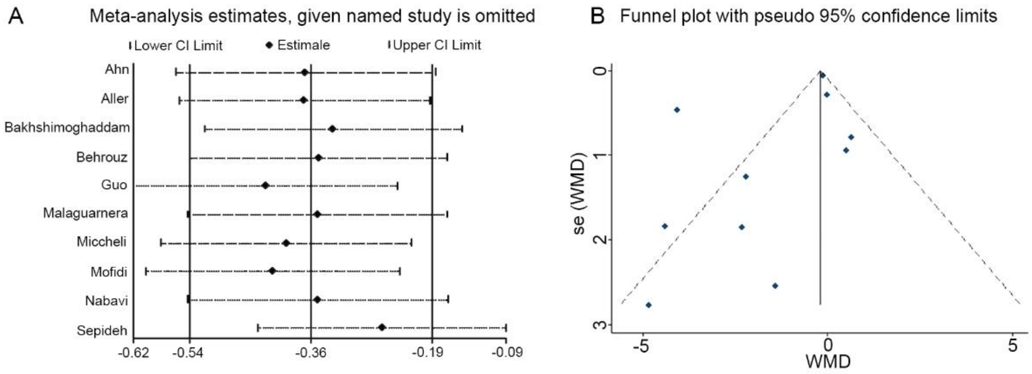

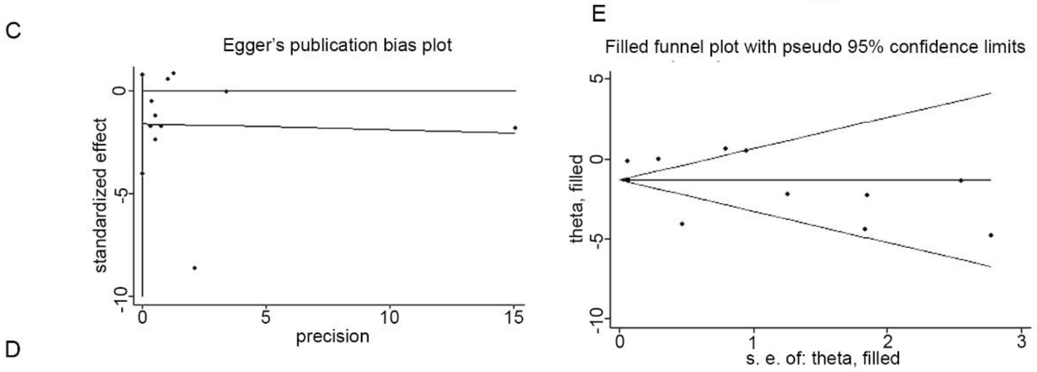

D

```
Egger's test

    Std_Eff |      Coef.   Std. Err.      t    P>|t|     [95% Conf. Interval]
------------+----------------------------------------------------------------
      slope |  -.0294895    .2096254   -0.14   0.892    -.5128867    .4539076
       bias |    -1.6104    1.042689   -1.54   0.161    -4.014845    .7940457
```

F

```
Meta-analysis

       |  Pooled      95% CI         Asymptotic      No. of
Method |     Est   Lower   Upper  z_value  p_value   studies
-------+----------------------------------------------------
Fixed  |  -0.196  -0.320  -0.071   -3.081    0.002      10
Random |  -1.316  -2.427  -0.205   -2.322    0.020

Test for heterogeneity: Q= 83.095 on 9 degrees of freedom (p= 0.000)
Moment-based estimate of between studies variance =  1.924

Trimming estimator: Linear
Meta-analysis type: Random-effects model

iteration |  estimate    Tn    # to trim     diff
----------+--------------------------------------
    1     |   -1.316     22        0           55
    2     |   -1.316     22        0            0

Note: no trimming performed; data unchanged

Filled
Meta-analysis

       |  Pooled      95% CI         Asymptotic      No. of
Method |     Est   Lower   Upper  z_value  p_value   studies
-------+----------------------------------------------------
Fixed  |  -0.196  -0.320  -0.071   -3.081    0.002      10
Random |  -1.316  -2.427  -0.205   -2.322    0.020

Test for heterogeneity: Q= 83.095 on 9 degrees of freedom (p= 0.000)
Moment-based estimate of between studies variance =  1.924
```

Figure S5: Supplementary data of insulin, showing the interpretation of single study (A), funnel plot (B), Egger's tests (C, D), trim-and-fill computation (E, F).

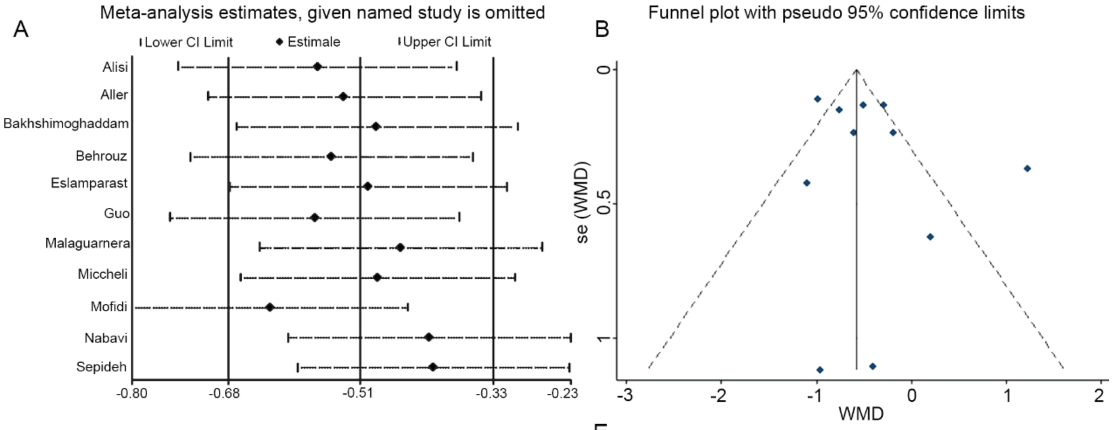

C

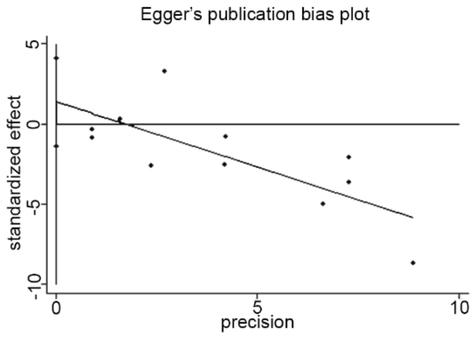

E

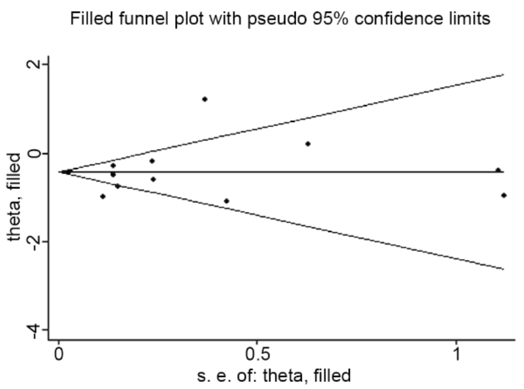

D

Egger's test

Std_Eff	Coef.	Std. Err.	t	P>|t|	[95% Conf.	Interval]
slope	-.8138002	.2404038	-3.39	0.008	-1.357631	-.2699691
bias	1.387598	1.215927	1.14	0.283	-1.363021	4.138216

F

```
Meta-analysis

       |  Pooled      95% CI         Asymptotic      No. of
Method |     Est    Lower    Upper  z_value  p_value   studies
-------+----------------------------------------------------
Fixed  |  -0.582  -0.699  -0.465   -9.768   0.000      11
Random |  -0.425  -0.735  -0.115   -2.688   0.007

Test for heterogeneity: Q= 48.244 on 10 degrees of freedom (p= 0.000)
Moment-based estimate of between studies variance =  0.166

Trimming estimator: Linear
Meta-analysis type: Random-effects model

iteration |  estimate     Tn     # to trim     diff
----------+--------------------------------------
    1     |   -0.425      29         0          66
    2     |   -0.425      29         0           0

Note: no trimming performed; data unchanged

Filled
Meta-analysis

       |  Pooled      95% CI         Asymptotic      No. of
Method |     Est    Lower    Upper  z_value  p_value   studies
-------+----------------------------------------------------
Fixed  |  -0.582  -0.699  -0.465   -9.768   0.000      11
Random |  -0.425  -0.735  -0.115   -2.688   0.007

Test for heterogeneity: Q= 48.244 on 10 degrees of freedom (p= 0.000)
Moment-based estimate of between studies variance =  0.166
```

Figure S6: Supplementary data of homeostasis model assessment- insulin resistance (HOMA-IR), showing the interpretation of single study (A), funnel plot (B), Egger's tests (C, D), trim-and-fill computation (E, F).

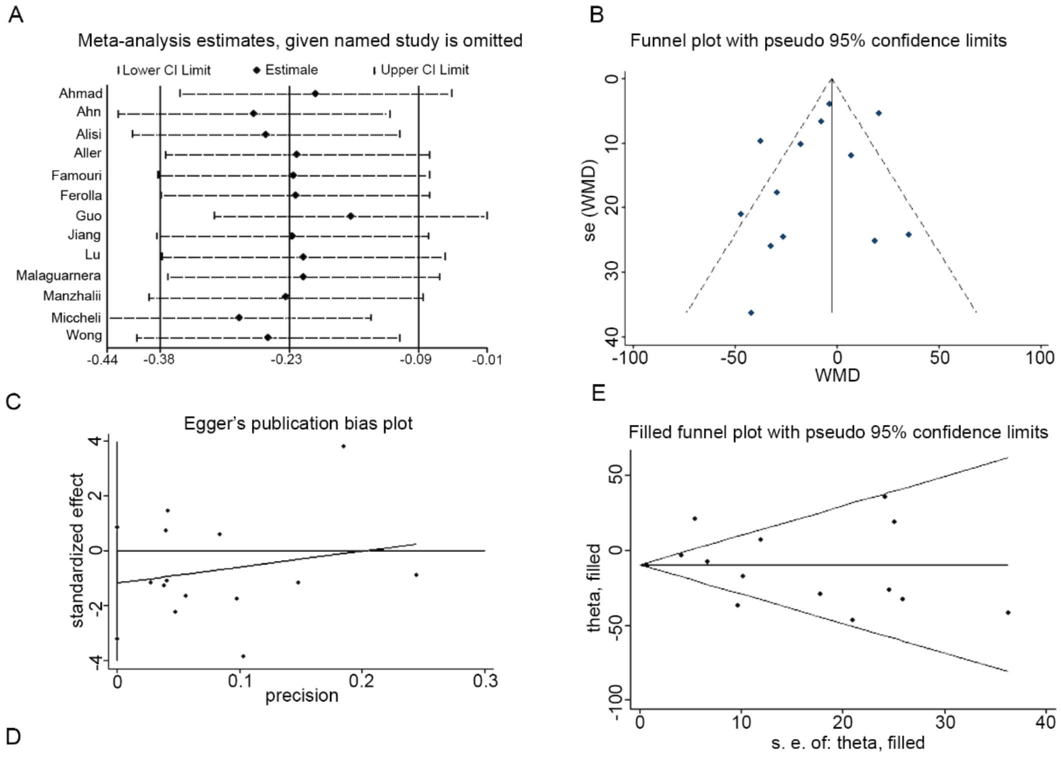

D

```
Egger's test

      Std_Eff |      Coef.   Std. Err.      t    P>|t|     [95% Conf. Interval]
--------------+------------------------------------------------------------------
        slope |    5.86498   8.433426     0.70   0.501    -12.69686    24.42683
         bias |  -1.165758   .9232586    -1.26   0.233    -3.197837    .8663205
```

F

```
Meta-analysis

       |  Pooled      95% CI         Asymptotic      No. of
Method |     Est    Lower   Upper  z_value  p_value   studies
-------+----------------------------------------------------
Fixed  |  -2.771   -7.737   2.194   -1.094   0.274      13
Random |  -9.598  -22.129   2.933   -1.501   0.133

Test for heterogeneity: Q= 47.762 on 12 degrees of freedom (p= 0.000)
Moment-based estimate of between studies variance = 296.836

Trimming estimator: Linear
Meta-analysis type: Random-effects model

iteration |  estimate    Tn     # to trim     diff
----------+--------------------------------------
    1     |   -9.598     39         0          91
    2     |   -9.598     39         0           0

Note: no trimming performed; data unchanged

Filled
Meta-analysis

       |  Pooled      95% CI         Asymptotic      No. of
Method |     Est    Lower   Upper  z_value  p_value   studies
-------+----------------------------------------------------
Fixed  |  -2.771   -7.737   2.194   -1.094   0.274      13
Random |  -9.598  -22.129   2.933   -1.501   0.133

Test for heterogeneity: Q= 47.762 on 12 degrees of freedom (p= 0.000)
Moment-based estimate of between studies variance = 296.836
```

Figure S7: Supplementary data of triglycerides (TG), showing the interpretation of single study (A), funnel plot (B), Egger's tests (C, D), trim-and-fill computation (E, F).

A

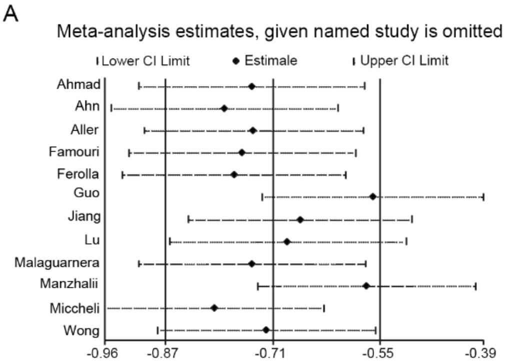

B

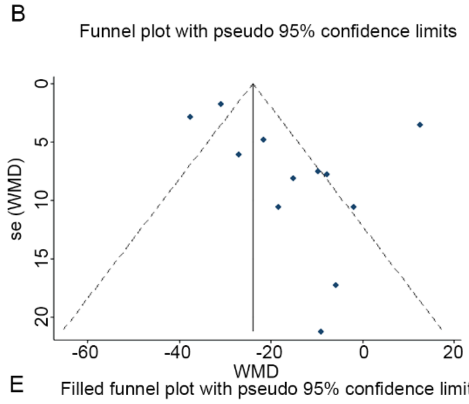

C

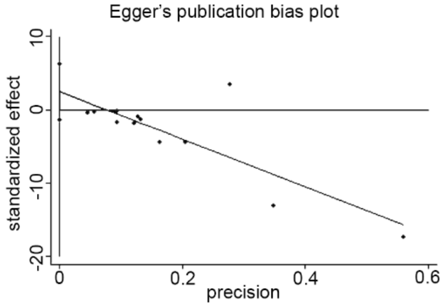

E

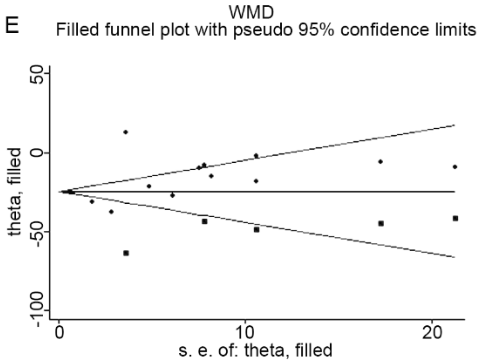

D

Egger's test

Std_Eff	Coef.	Std. Err.	t	P>|t|	[95% Conf.	Interval]
slope	-32.52605	7.330475	-4.44	0.001	-48.85937	-16.19273
bias	2.494808	1.708969	1.46	0.175	-1.313014	6.302629

F

```
Meta-analysis

       |  Pooled      95% CI         Asymptotic      No. of
Method |     Est   Lower   Upper  z_value  p_value   studies
-------+----------------------------------------------------
Fixed  | -23.999 -26.426 -21.572  -19.382    0.000      12
Random | -15.375 -26.501  -4.250   -2.709    0.007

Test for heterogeneity: Q= 155.233 on 11 degrees of freedom (p= 0.000)
Moment-based estimate of between studies variance = 310.327

Trimming estimator: Linear
Meta-analysis type: Random-effects model

iteration |  estimate    Tn    # to trim     diff
----------+--------------------------------------
    1     |  -15.375     42         1          78
    2     |  -20.694     58         3          32
    3     |  -22.965     63         4          10
    4     |  -25.120     68         5          10
    5     |  -25.469     68         5           0

Filled
Meta-analysis

       |  Pooled      95% CI         Asymptotic      No. of
Method |     Est   Lower   Upper  z_value  p_value   studies
-------+----------------------------------------------------
Fixed  | -28.834 -31.082 -26.585  -25.132    0.000      17
Random | -24.490 -35.438 -13.542   -4.384    0.000

Test for heterogeneity: Q= 270.640 on 16 degrees of freedom (p= 0.000)
Moment-based estimate of between studies variance = 431.164
```

Figure S8: Supplementary data of total cholesterol (TC), showing the interpretation of single study (A), funnel plot (B), Egger's tests (C, D), trim-and-fill computation (E, F).

A

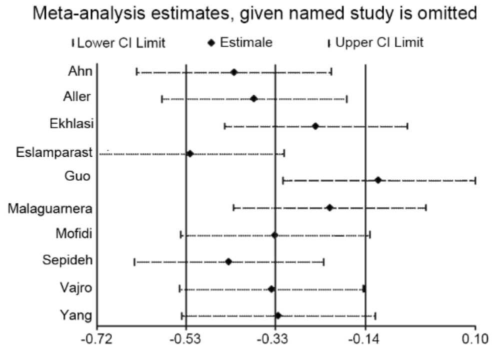

B

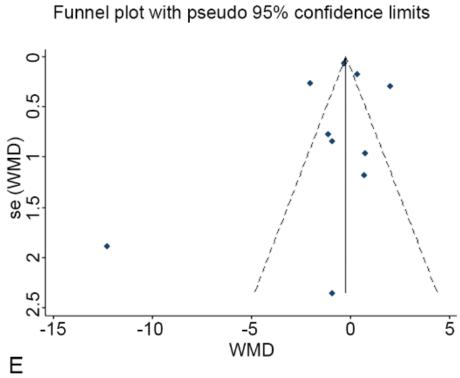

C

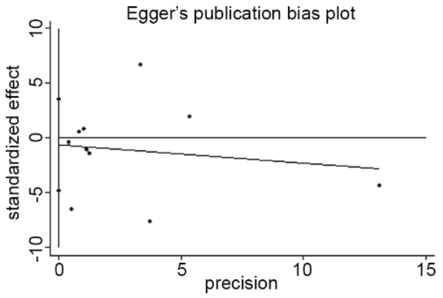

E

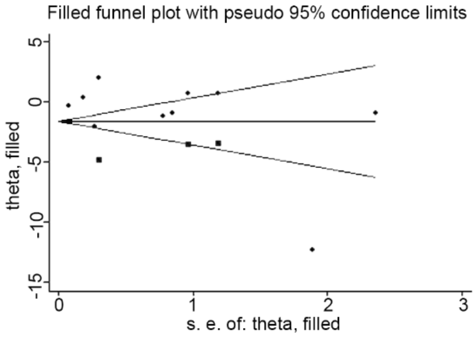

D

Egger's test

Std_Eff	Coef.	Std. Err.	t	P>|t|	[95% Conf.	Interval]
slope	-.1666514	.3772674	-0.44	0.670	-1.036632	.7033288
bias	-.6229142	1.811553	-0.34	0.740	-4.800362	3.554533

F

```
Meta-analysis

       |  Pooled      95% CI         Asymptotic      No. of
Method |     Est   Lower   Upper  z_value  p_value   studies
-------+----------------------------------------------------
Fixed  |  -0.250  -0.379  -0.121   -3.795    0.000        10
Random |  -0.649  -1.565   0.266   -1.390    0.165

Test for heterogeneity: Q= 156.674 on 9 degrees of freedom (p= 0.000)
Moment-based estimate of between studies variance =  1.506

Trimming estimator: Linear
Meta-analysis type: Random-effects model

iteration |  estimate    Tn    # to trim    diff
----------+--------------------------------------
    1     |   -0.649     31        1          55
    2     |   -0.997     37        2          12
    3     |   -1.193     41        3           8
    4     |   -1.383     41        3           0

Filled
Meta-analysis

       |  Pooled      95% CI         Asymptotic      No. of
Method |     Est   Lower   Upper  z_value  p_value   studies
-------+----------------------------------------------------
Fixed  |  -0.482  -0.607  -0.356   -7.518    0.000        13
Random |  -1.602  -2.695  -0.509   -2.872    0.004

Test for heterogeneity: Q= 390.698 on 12 degrees of freedom (p= 0.000)
Moment-based estimate of between studies variance =  3.220
```

Figure S9: Supplementary data of tumor necrosis factor – alpha (*Tnf-α*), showing the interpretation of single study (A), funnel plot (B), Egger's tests (C, D), trim-and-fill computation (E, F).

Supplemental table 2: Evidence quality assessment

Question: Should probiotics be used for NAFLD?

Item: BMI

Quality assessment							No of patients		Effect		Quality	Importance
**No of studies**	**Design**	**Risk of bias**	**Inconsistency**	**Indirectness**	**Imprecision**	**Other considerations**	**Probiotics**	**Control**	**Relative (95% CI)**	**Absolute**		
**BMI (Better indicated by lower values)**												
15	randomised trials	serious[1]	serious[2]	no serious indirectness	no serious imprecision	strong association[3]	410	408	-	MD 0.56 lower (0.66 to 0.47 lower)	⊕⊕⊕O MODERATE	IMPORTANT

[1] Studies included possess several bias

[2] Studies included reported inconsistency results

[3] Studies included are more than 10

Item: Liver function

Quality assessment							No of patients		Effect		Quality	Importance
**No of studies**	**Design**	**Risk of bias**	**Inconsistency**	**Indirectness**	**Imprecision**	**Other considerations**	**Probiotics**	**Control**	**Relative (95% CI)**	**Absolute**		
**ALT (Better indicated by lower values)**												
20	randomised trials	serious[1]	no serious inconsistency	no serious indirectness	no serious imprecision	very strong association[2]	561	555	-	MD 13.4 lower (17.03 to 9.77 lower)	⊕⊕⊕⊕ HIGH	CRITICAL
**AST (Better indicated by lower values)**												
17	randomised trials	serious[1]	serious[3]	no serious indirectness	no serious imprecision	strong association[4]	499	493	-	MD 12.2 lower (12.86	⊕⊕⊕O MODERATE	CRITICAL

										to 11.53 lower)		
**GGT (Better indicated by lower values)**												
7	randomised trials	serious[5]	serious[3]	no serious indirectness	no serious imprecision	none	248	240	-	MD 6.44 lower (7.46 to 5.41 lower)	⊕⊕OO LOW	CRITICAL
**LSM (Better indicated by lower values)**												
4	randomised trials	serious[5]	no serious inconsistency	no serious indirectness	Serious[5]	none	99	103	-	MD 0.65 lower (0.76 to 0.55 lower)	⊕⊕OO LOW	CRITICAL
**Hepatic steatosis (Better indicated by lower values)**												
4	randomised trials	serious[5]	no serious inconsistency	no serious indirectness	Serious[5]	none	131	128	-	not pooled	⊕⊕OO LOW	CRITICAL

[1] Studies included possess several bias

[2] Studies included are more than 20

[3] Studies included reported inconsistency results

[4] Studies included are more than 10

[5] Studies included are less than 5

Item: Glycemic indices

**Quality assessment**							**No of patients**		**Effect**		**Quality**	**Importance**
**No of studies**	**Design**	**Risk of bias**	**Inconsistency**	**Indirectness**	**Imprecision**	**Other considerations**	**Probiotics**	**Control**	**Relative (95% CI)**	**Absolute**		
**FBS (Better indicated by lower values)**												
13	randomised trials	serious[1]	serious[2]	no serious indirectness	no serious imprecision	strong association[3]	346	347	-	MD 2.52 lower (3.7 to 1.34 lower)	⊕⊕⊕O MODERATE	IMPORTANT

**Insulin (Better indicated by lower values)**												
10	randomised trials	serious[1]	no serious inconsistency	no serious indirectness	no serious imprecision	strong association[3]	271	273	-	MD 0.2 lower (0.32 to 0.07 lower)	⊕⊕⊕⊕ HIGH	
**GLP-1 (Better indicated by lower values)**												
2	randomised trials	serious[1]	no serious inconsistency	no serious indirectness	serious[4]	none	37	38	-	MD 1.37 higher (1.24 to 1.5 higher)	⊕⊕OO LOW	IMPORTANT
**HOMA-IR (Better indicated by lower values)**												
12	randomised trials	serious[1]	serious[2]	no serious indirectness	no serious imprecision	strong association[3]	317	317	-	MD 0.58 lower (0.7 to 0.47 lower)	⊕⊕⊕O MODERATE	IMPORTANT

[1] Studies included possess several bias

[2] Studies included reported inconsistency results

[3] Studies included are more than 10

[4] Studies included are less than 5

Item: Lipid profiles

Quality assessment							No of patients		Effect		Quality	Importance
No of studies	Design	Risk of bias	Inconsistency	Indirectness	Imprecision	Other considerations	Probiotics	Control	Relative (95% CI)	Absolute		
**HDL-C (Better indicated by lower values)**												
9	randomised trials	serious[1]	serious[2]	no serious indirectness	no serious imprecision	none	243	240	-	MD 1.36 higher (0.01 lower to 2.73 higher)	⊕⊕OO LOW	IMPORTANT
**LDL-C (Better indicated by lower values)**												

8	randomised trials	serious[1]	serious[2]	no serious indirectness	no serious imprecision	none	212	208	-	MD 0.31 higher (3.52 lower to 4.15 higher)	⊕⊕OO LOW	IMPORTANT
**Triglyceride (Better indicated by lower values)**											
13	randomised trials	serious[1]	serious[2]	no serious indirectness	no serious imprecision	strong association[3]	383	383	-	MD 2.77 lower (7.74 lower to 2.19 higher)	⊕⊕⊕O MODERATE	IMPORTANT
**Cholesterol (Better indicated by lower values)**											
12	randomised trials	serious[1]	serious[2]	no serious indirectness	no serious imprecision	strong association[3]	361	361	-	MD 24 lower (26.43 to 21.57 lower)	⊕⊕⊕O MODERATE	IMPORTANT

[1] Studies included possess several bias

[2] Studies included reported inconsistency results

[3] Studies included are more than 10

Item: Inflammation factors

**Quality assessment**							**No of patients**		**Effect**		**Quality**	**Importance**
**No of studies**	**Design**	**Risk of bias**	**Inconsistency**	**Indirectness**	**Imprecision**	**Other considerations**	**Probiotics**	**Control**	**Relative (95% CI)**	**Absolute**		
**TNF-a (Better indicated by lower values)**												
10	randomised trials	serious[1]	serious[2]	no serious indirectness	no serious imprecision	reporting bias strong association[4]	239	240	-	MD 0.25 lower (0.38 to 0.12 lower)	⊕⊕OO LOW	IMPORTANT
**IL-6 (Better indicated by lower values)**												
4	randomi	seriou	serious[2]	no serious	Serious[4]	none	95	100	-	MD	⊕OO	IMPORTA

	sed trials	s[1]		indirectness						0.08 lower (0.37 lower to 0.21 higher)	O VERY LOW	NT
**CRP (Better indicated by lower values)**												
4	randomised trials	serious[1]	no serious inconsistency	no serious indirectness	Serious[4]	none	117	111	-	MD 1.27 lower (2.1 to 0.44 lower)	⊕⊕OO LOW	IMPORTANT

[1] Studies included possess several bias

[2] Studies included reported inconsistency results

[3] Studies included are more than 10

[4] Studies included are less than 5

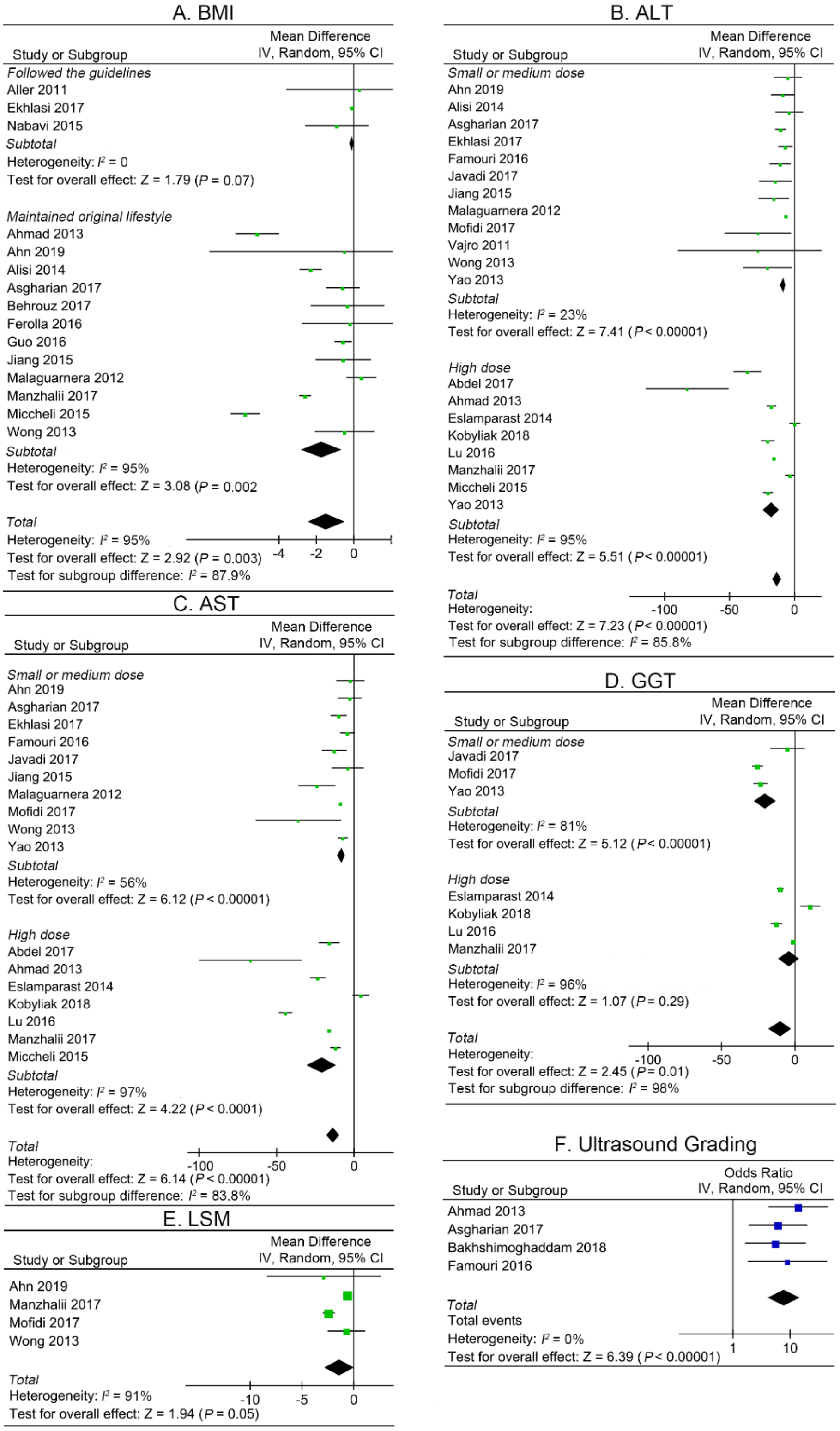

Figure S10: Forest plots of comparison for the effects of probiotics in NAFLD patients, showing (A) body mass index (BMI), (B) alanine aminotransferase (ALT), (C) aspartate transaminase (AST), (D) gamma -glutamyl transpeptidase (GGT), and subgroup analyses by probiotic strains of each index.
